# Studies on the Kinetics of the CH + H_2_ Reaction and Implications for the Reverse Reaction, ^3^CH_2_ + H

**DOI:** 10.1021/acs.jpca.2c08097

**Published:** 2023-03-01

**Authors:** Mark A. Blitz, Lavinia Onel, Struan H. Robertson, Paul W. Seakins

**Affiliations:** † School of Chemistry, University of Leeds, Leeds LS2 9JT, U.K.; ‡ NCAS, University of Leeds, Leeds LS2 9JT, U.K.; § Dassault Systemes, 334 Science Park, Cambridge CB2 0WN, U.K.

## Abstract

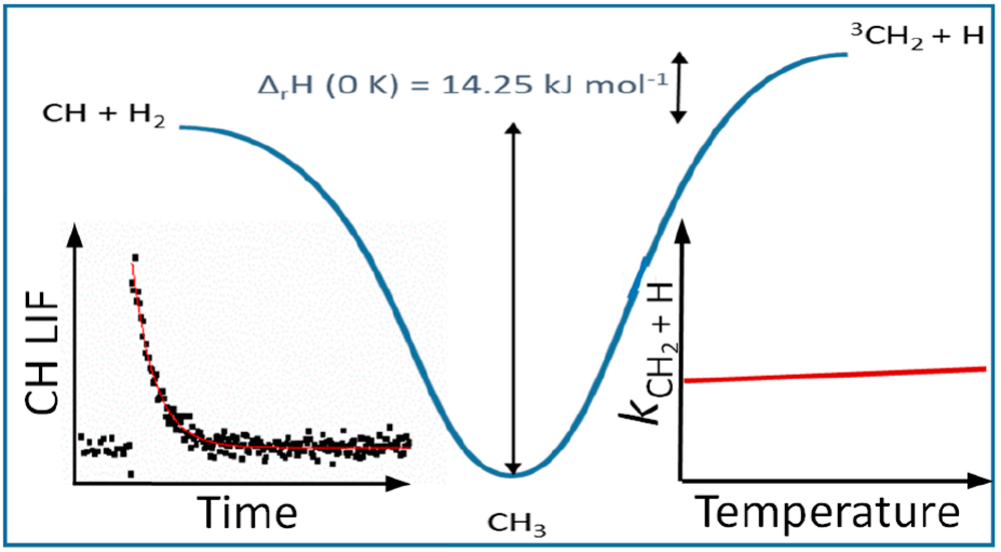

The reaction of CH radicals with H_2_ has been studied by the use of laser flash photolysis, probing CH decays under pseudo-first-order conditions using laser-induced fluorescence (LIF) over the temperature range 298–748 K at pressures of ∼5–100 Torr. Careful data analysis was required to separate the CH LIF signal at ∼428 nm from broad background fluorescence, and this interference increased with temperature. We believe that this interference may have been the source of anomalous pressure behavior reported previously in the literature (BrownswordR. A.; J. Chem. Phys.1997, 106, 7662−7677). The rate coefficient *k*_1_ shows complex behavior: at low pressures, the main route for the CH_3_* formed from the insertion of CH into H_2_ is the formation of ^3^CH_2_ + H, and as the pressure is increased, CH_3_* is increasingly stabilized to CH_3_. The kinetic data on CH + H_2_ have been combined with experimental shock tube data on methyl decomposition and literature thermochemistry within a master equation program to precisely determine the rate coefficient of the reverse reaction, ^3^CH_2_ + H → CH + H_2_. The resulting parametrization is *k*_CH_2_+H_(*T*) = (1.69 ± 0.11) × 10^–10^ × (*T*/298 K)^(0.05±0.010)^ cm^3^ molecule^–1^ s^–1^, where the errors are 1σ.

## Introduction

The methylidene radical, CH(X^2^Π) is a key intermediate in combustion,^1^ in reduced atmospheres of planets and moons such as Jupiter and Titan,^2^ and in the interstellar medium.^3^ The reactivity of CH is such that it can insert into strong bonds, initiating the “prompt” formation of NO_*x*_ when reacting with N_2_^4^ and undergoing a number of insertion/elimination reactions with alkanes, alkenes, and alkynes.^5−11^ There have been a number of studies of CH kinetics; laser flash photolysis studies have tended to use the multiphoton photolysis of bromoform, CHBr_3_, to generate CH,^2−9^ whereas flow tube studies have used metal atom stripping of Br from CHBr_3_ to generate CH.^12^ Most studies use laser-induced fluorescence at ∼431 nm to probe the CH concentration as a function of time, but chemiluminescence from the CH + O_2_ reaction has also been used.^13^ Given the high endothermicity of CH (Δ_f_*H*°(0 K) = 592.837 ± 0.097 kJ mol^–1^),^14^ many CH reactions have multiple product channels, and several groups have determined quantitative product yields for key CH reactions.^6,10,15^

**Figure 1:**
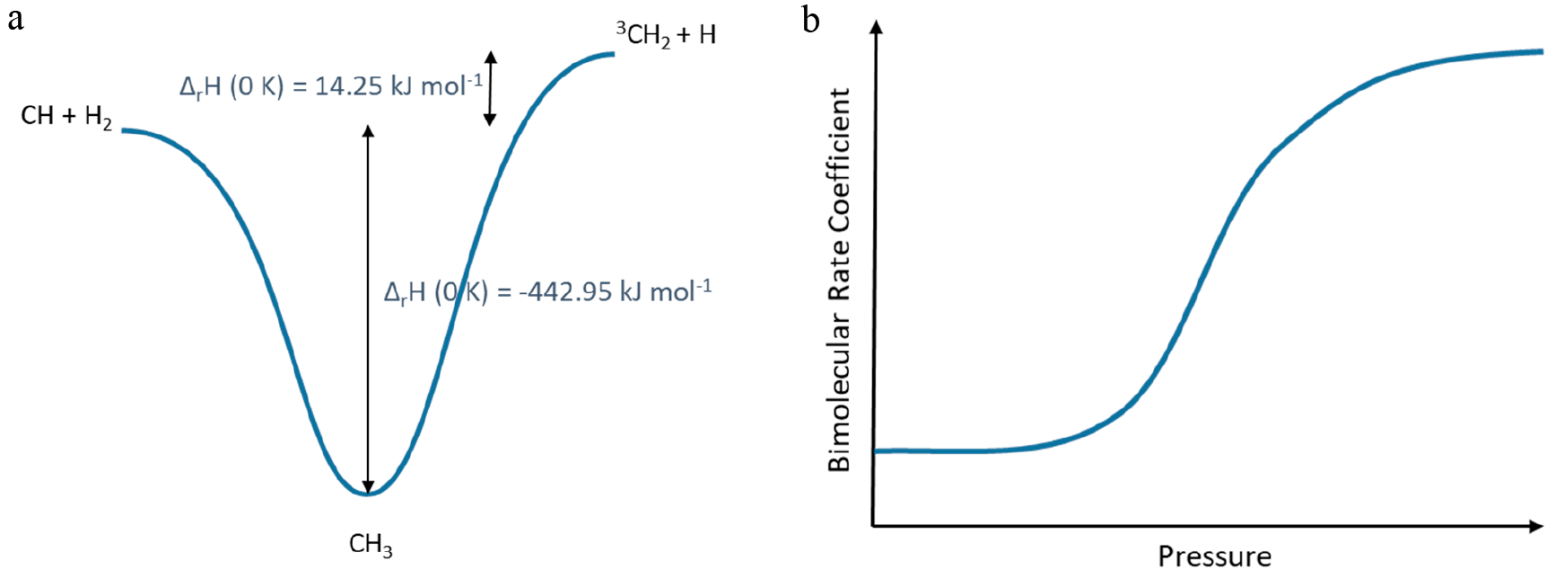
(a) Schematic potential energy surface for reaction 1. Values for Δ_r_*H*(0 K) were taken from the Active Thermochemical Tables (ATcT).^14^ (b) Schematic showing the expected pressure dependence of reaction 1. At low pressure a limiting value is obtained corresponding to the formation of ^3^CH_2_ + H. As the pressure increases, stabilization into the methyl well enhances the rate coefficient.

An important reaction of CH in combustion and in the atmospheres of the giant planets is the reaction with H_2_ (reaction 1):^16−20^
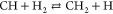
±1The CH + H_2_ reaction is thought to proceed via an insertion forming chemically activated methyl radicals, CH_3_*, that can either be stabilized or eliminate a hydrogen atom to form ground-state methylene, ^3^CH_2_, as the coproduct:
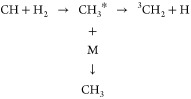
The thermodynamics of the system is well-established, and the potential energy surface (PES) has been calculated by a number of groups (see, e.g., refs (21) and (22)). A schematic of the PES is shown in Figure 1a. The kinetics of the CH + H_2_ reaction is expected to show an S-shaped pressure dependence, as shown in Figure 1b; at low pressures no CH_3_* is stabilized, and the loss of CH is determined by the competition between the forward and backward unimolecular decompositions of CH_3_*. As the pressure increases, CH_3_* will start to be stabilized into the CH_3_ well, and the overall rate coefficient will increase with pressure until it reaches a limit where stabilization outcompetes redissociation. This high-pressure rate coefficient reflects the rate coefficient for CH_3_* formation.

Reaction 1 was extensively studied by Brownsword et al.^16^ At low temperatures, the system behaved as expected, but at the highest temperatures of the study (584 and 744 K) a negative pressure dependence was observed at lower pressures (5–100 Torr). Brownsword et al. suggested that at high temperatures, where a majority of the reagent population is above the threshold to form ^3^CH_2_ + H and hence a majority of CH_3_* is also above this threshold, collisional deactivation could bring a significant proportion of energized CH_3_* below the threshold for ^3^CH_2_ + H but above the CH + H_2_ asymptote, promoting re-formation of reagents and reducing the rate coefficient as a function of pressure. As the pressure increases further, stabilization to CH_3_ dominates, and the rate coefficient reverts to the expected behavior, increasing with pressure.

The well-known thermodynamics of the system means that the kinetics of the reverse reaction, ^3^CH_2_ + H (reaction −1), can be determined from studies on CH + H_2_ kinetics. In contrast to the relatively straightforward way that the forward reaction can be studied (at least at lower temperatures), the reverse reaction is experimentally challenging, requiring the generation of two radical species. If the reaction is carried out under pseudo-first-order conditions ([H] ≫ [^3^CH_2_]), the absolute concentration of hydrogen atoms needs to be known; for second-order kinetics, both radical concentrations need to be known. ^3^CH_2_ is also not that easy to detect, with most studies using laser magnetic resonance (LMR) or mass spectrometry. These challenges are reflected in the reported values for *k*_–1_ summarized in Table 1.

**Table 1:** Summary of Previous Kinetics Studies on the ^3^CH_2_ + H Reaction

authors, year	temperature range/K	pressure range	*k*_300K_/cm^3^ molecule^–1^ s^–1^	*k*(*T*)/cm^3^ molecule^–1^ s^–1^	method
Fulle and Hippler, 1997^20^	185–800	1–160 bar	2.2 × 10^–10^	(2.19 × 10^–10^)(*T*/298)^0.32^	application of equilibrium to studies of the reverse reaction
Rohrig et al., 1997^23^	2200–2600	1 bar		2.34 × 10^–10^	indirect measurement from shock tube study of CH + O_2_
Devriendt et al., 1995^24^	300–1000	2 Torr	4.9 × 10^–10^	5.2 × 10^–11^ exp(5610/*RT*)	mass spectrometry: rate coefficient measured relative to CH_2_ + O
Boullart and Peeters, 1992^25^	300	2 Torr	2.7 × 10^–10^	n.a.	mass spectrometry: rate coefficient measured relative to CH_2_ + O
Bohland et al., 1987^26^	298	1–1.6 Torr	1.83 × 10^–10^	n.a.	discharge flow, ^3^CH_2_ monitored with laser magnetic resonance
Zabernack et al., 1986^27^	300–700		1.4 × 10^–10^	4.7 × 10^–10^ exp(−3080/*RT*)	application of equilibrium to studies of the reverse reaction

Although ^3^CH_2_ has a very high enthalpy of formation (Δ_f_*H*°(0 K) = 391.054 ± 0.096 kJ mol^–1^),^14,28^ it is relatively unreactive and much less reactive than the first excited state, ^1^CH_2_, which is only 37.66 kJ mol^–1^ higher in energy, not undergoing any of the insertion/elimination reactions that characterize ^1^CH_2_ chemistry (see, e.g., ref (29)). The reaction of ^3^CH_2_ with H is a notable exception in terms of reactivity, and although there is a wide variation in the magnitude of *k*_–1_ and even some uncertainty in the direction of its temperature dependence, the rate coefficient is relatively high (∼0.8^30^ to 5^24^ × 10^–10^ cm^3^ molecule^–1^ s^–1^). Hence in environments with high [H] such as flames^31,32^ and the upper regions of the atmospheres of the giant planets,^33^reaction −1 can convert relatively unreactive ^3^CH_2_ into reactive CH species. For combustion chemistry, both Hughes et al.^31^ and Glarborg et al.^32^ highlighted the important role of reaction −1, the scatter of the current literature data, and the need for further studies.

In this paper we present new experimental data on the reaction of CH + H_2_ with a particular focus on resolving the abnormal pressure dependence at high temperatures and low pressures. We then combine our data with literature measurements on reaction 1 and test the dataset for consistency using the master equation program MESMER.^34−36^ Given the well-defined thermochemistry for reactions 1 and −1, precise predictions of *k*_–1_ based on the consensus data of *k*_1_ can be made.

## Methods

### Experimental Section

Reaction 1 was studied in a conventional slow-flow laser flash photolysis system with time-resolved detection of CH by laser-induced fluorescence (LIF) under pseudo-first-order conditions. Details on previous studies can be found in McKee et al.^6^ and Blitz et al.^15,37^ The CH precursor, bromoform (Sigma-Aldrich, 98% purity) was stored as dilute mixtures (argon) in glass bulbs and metered using calibrated mass flow controllers. Precursor gas mixtures, H_2_ (BOC, >99.99%), and argon bath gas were combined in a mixing manifold and flowed through a metal reaction cell. Additionally, a few experiments were carried out with helium as the bath gas. Temperatures were recorded with calibrated thermocouples, and pressures in the cell were determined via capacitance manometers.

Bromoform was photolyzed by an excimer laser at 248 nm. CH production occurs via a multiphoton absorption process, and several other species such as H and CHBr_*x*_ will be formed in the photolysis:
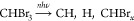
2Typical excimer fluences were 50 mJ cm^–2^ pulse^–1^, and the excimer laser was operated at 10 Hz, which was sufficient for replenishment of a fresh gas mix for each laser shot.

CH was probed via LIF at 428 nm (the R_1_(4) line of the A ← X transition) generated from a YAG (Quantel 850, 355 nm, 10 Hz)-pumped dye laser (Sirah, operating on Stilbene 420 dye), and resonant fluorescence was observed perpendicular to the axes of both the photolysis and probe lasers with a microchannel plate photomultiplier. The time delay between the photolysis and probe pulses was varied in order to build up a decay curve.

**Figure 2:**
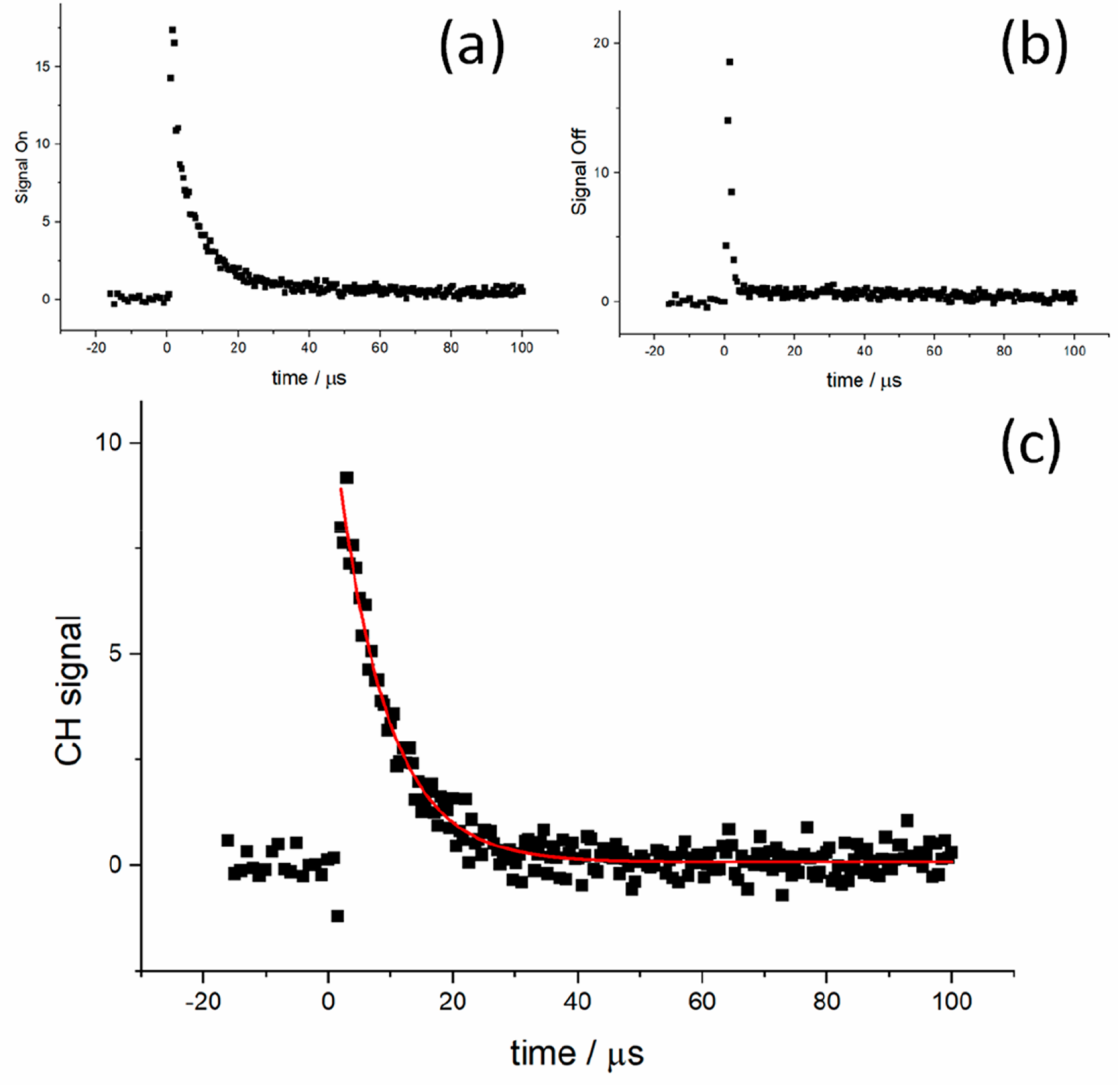
Elimination of interference. (a) Total signal recorded at ∼428 nm (R_1_(4) line), CH and background signal. (b) Signal recorded with the dye laser moved 0.17 nm, i.e., off the R_1_(4) CH line but with a very similar background signal. (c) Subtraction of the two traces yields the CH-only signal. The red line is an exponential fit to the data.

At close to time zero, CH* emission was observed that is a result of the multiphoton dissociation of CHBr_3_. In addition, as the temperature was increased a structureless spectrum was observed between 425–440 nm, possibly due to LIF from a CHBr_*x*_ species. This emission swamps the strong CH LIF line at 431 nm that is normally used to monitor CH but has less impact on the R_1_(4) line at 428 nm. We experimented with a narrow-band filter (430 ± 5 nm), but this had limited effect, as it passed a significant amount of both the CH* emission and the background LIF. In order to account for both the spontaneous fluorescence from CH* and LIF from the unknown species, two decay traces were recorded for each condition, first with the probe laser tuned to the peak of the R_1_(4) line (recording emission from CH*, CH, and the unknown species) and then with the laser moved by ∼0.17 nm, sufficient to move off the R_1_(4) line, but without any significant change in the LIF signal from the unknown species. Subtraction of the two decay profiles yielded the CH LIF decay. An example of the process is shown in Figure 2.

The system was studied under pseudo-first-order conditions with [H_2_] ≫ [CH]_0_. Under these conditions, the resulting CH decay curves should be exponential (as shown in Figure 2c) with the pseudo-first-order rate coefficient, *k*_obs_, being given by
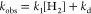
3where *k*_1_ is the pressure-dependent bimolecular rate coefficient and *k*_d_ is an effective first-order rate coefficient that accounts for the reaction of CH with bromoform and diffusion out of the observation region.

**Figure 3:**
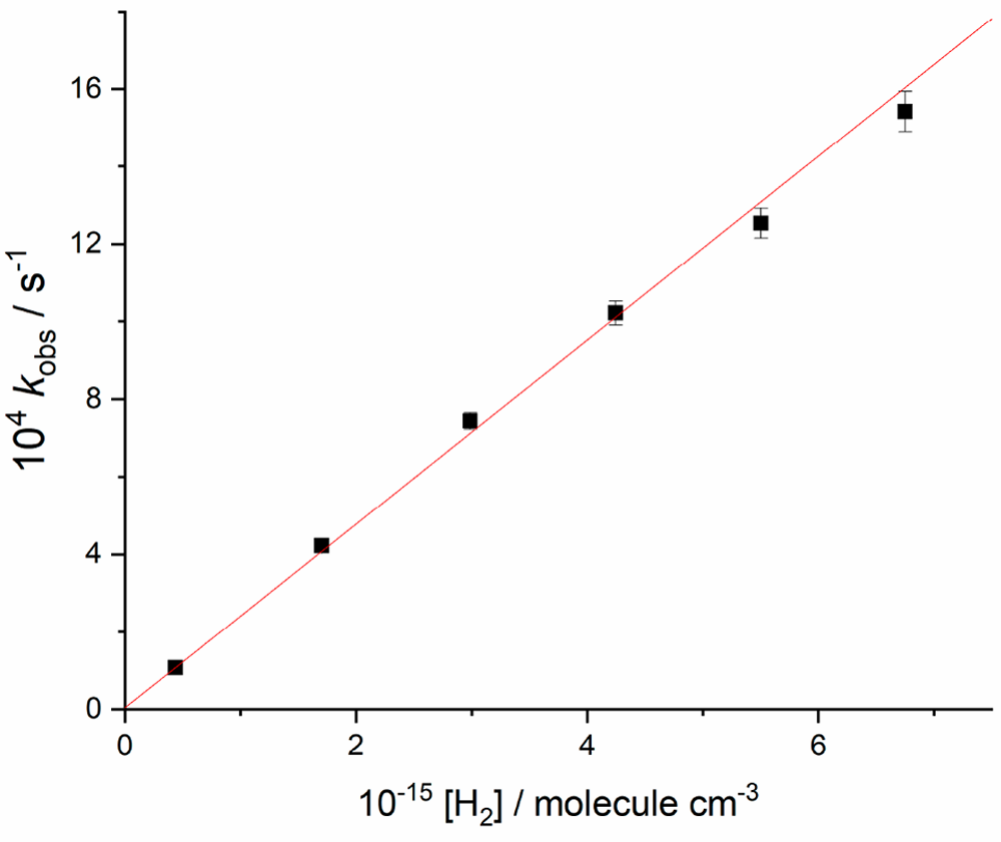
Bimolecular plot at 748 K and 70.3 Torr total pressure of argon. A weighted linear fit to the data gives *k*_1_ = (2.37 ± 0.05) × 10^–11^ cm^3^ molecule^–1^ s^–1^, where the error reported is the statistical error at the 1σ level.

**Figure 4:**
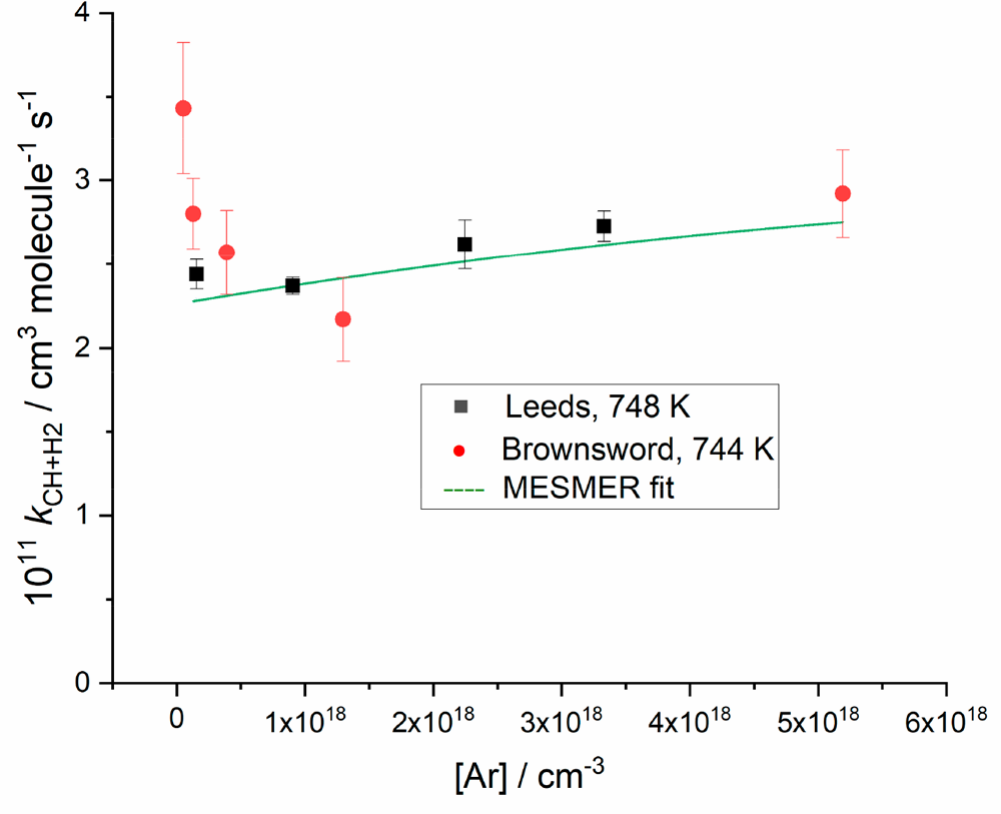
Example of the pressure dependence of *k*_1_ recorded at the temperature of ∼744 K. The figure shows data from this work (black ■) and Brownsword et al.^16^ (red ●).

Figure 3 shows a typical bimolecular plot of *k*_obs_ versus [H_2_], the gradient of which is *k*_1_. Experiments were repeated for a range of pressures at each temperature, and checks were made to ensure the invariance of the rate coefficients with photolysis laser energy or repetition rate. Figure 4 shows the dependence of *k*_1_ as a function of pressure at ∼744 K. The data are tabulated in Table S1. The errors reported for the bimolecular rate coefficients in this article are the statistical errors at the 1σ level from the bimolecular plots. We suggest that these errors should be combined with an estimated 10% systematic error to give the overall error. These errors are reported in Table S1.

### MESMER Analysis

The master equation program MESMER was used to analyze the data obtained in this study and some literature data. Details on the MESMER program can be found in ref (34), and applications to analysis of literature data can be found in refs (36) and (38). Simulations using MESMER were undertaken to explore whether it was possible to reproduce the observations of Brownsword et al.^16^

MESMER contains a data-fitting routine based on Marquardt least-squares analysis. In the current analysis a variety of parameters were varied. The energy transfer parameter, ⟨Δ*E*⟩_d_ (the average transferred in a downward direction), was assigned a temperature dependence of the form *A*(298 K) × *T*^*n*^, and values of *A* and *n* could be set for each bath gas, allowing us to directly compare experiments carried out in He and Ar.

The first data analysis was limited to experimental studies on the CH + H_2_ reaction. The datasets used were those from McIlroy and Tully,^39^ Fulle and Hippler,^20^ Brownsword et al.^16^ and Zabarnick et al.^27^ A second stage in the analysis was to combine high-temperature shock tube data on CH_3_ decomposition. A number of studies have looked at CH_3_ decomposition, probing both the loss of CH_3_ and product formation. The study by Vasudevan et al.^40^ monitored both CH and OH productions in the presence of oxygen. OH production was associated with the conversion of ^3^CH_2_ and H to OH via the well-studied reaction of ^3^CH_2_ with oxygen^41^ and the H + O_2_ reaction. However, CH will also react with O_2_ to generate OH, so OH production is not a unique signature of ^3^CH_2_ production.

## Results and Discussion

### Experimental Studies on the Reaction of CH with H_2_

Figure 4 shows an example of the pressure-dependent bimolecular rate coefficients. In their work, Brownsword et al.^16^ had observed significant *decreases* in the bimolecular rate coefficient *k*_1_ with pressure at low pressures (e.g., (3.43 ± 0.39) × 10^–11^ cm^3^ molecule^–1^ s^–1^ to (2.17 ± 0.25) × 10^–11^ cm^3^ molecule^–1^ s^–1^ from 4 to 100 Torr at 744 K; red points in Figure 4). Brownsword et al. carefully considered possible errors and uncertainties in their experiments but concluded that the effects were real. They suggested that a negative pressure effect could occur at high temperatures, where initial collisions could reduce the energy of CH_3_* below the exit channel for ^3^CH_2_ + H but still allowing redissociation to reagents and called for further investigations via a master equation approach.

We do not see a negative dependence with pressure over a similar pressure regime to that studied by Brownsword et al. (although they did go to slightly lower pressures), but there are some hints of unusual behavior at our very lowest pressures. We believe that this could be due to nonthermal ro-vibrational distributions in the photolytically produced CH. We carried out a range of calculations using MESMER but could not simulate any negative pressure effects even though we explored a wide range of conditions. Additionally, we constructed a three-state methyl model based on the hypothesis of Brownsword et al. considering CH_3_** (enough energy to form ^3^CH_2_ + H), CH_3_* (enough energy to dissociate back to reagents), and stabilized CH_3_. Using this model, it is indeed possible to simulate the behavior observed by Brownsword et al., but only if unrealistic parameters which cannot be included in a master equation approach (e.g., redissociation to reagents is slower for CH_3_** than CH_3_*) are used. Further details are given in section 2 of the Supporting Information. We suggest that the unusual behavior observed by Brownsword et al. is due to the prompt fluorescence or rotational relaxation not being fully accounted for, but there may be other possible explanations.

In general our data on reaction 1 are in good agreement with previous work: we observe the same trends of a positive temperature dependence in *k*_1_ at low pressure associated with the positive enthalpy of reaction to form ^3^CH_2_ + H and a positive pressure dependence at a fixed temperature as more CH_3_* is stabilized. A quantitative comparison with the existing literature is presented in the next section.

### MESMER Analysis

Our initial fits were based solely on the CH + H_2_ datasets. First, we fitted the data floating both the thermochemistry of reaction 1 and the energy transfer parameters. The resulting fits to the CH + H_2_ were reasonable (errors are 1σ), but the thermochemistry of the reaction, while in agreement with the literature, was not well-defined (Fit 1 in Table 2); hence, there are corresponding uncertainties in the kinetic parameters for reaction −1. An equally good fit, but with precise data on the kinetics of reaction −1, can be obtained by constraining the thermochemistry of reaction 1 to the values presented in ATcT. The parameters for this fit are shown as Fit 2 in Table 2. The reaction enthalpy in ATcT is Δ_r_*H*°(0 K) = 14.25 ± 0.11 kJ mol^–1^,^14,28,42^ where the error comes from the full covariance matrix at the 95% confidence limit. The individual uncertainties for CH and ^3^CH_2_ are low (∼0.1 kJ mol^–1^), and the recommended value is based on a wide range of provenance.

**Table 2:** Fitting Parameters for the CH + H_2_/CH_3_/^3^CH_2_ + H System (Reported Errors Are 1σ)

parameter	fit 1: CH + H_2_ data only; Δ_r_*H*_1_(0 K) floated	fit 2: CH + H_2_ Δ_r_*H*_1_(0 K) fixed to ATcT value	fit 3: CH + H_2_ and selected CH_3_ data; Δ_r_*H*_1_(0 K) fixed to ATcT value	fit 4: CH + H_2_ and selected CH_3_ data; extended *T* dependence of ⟨Δ*E*⟩_d_(*T*)
*A*_⟨Δ*E*⟩_d__ (Ar)/cm^–1^	42.3 ± 5.6	48.9 ± 2.5	50.6 ± 2.6	54.8 ± 2.9
*A*_⟨Δ*E*⟩_d__ (He)/cm^–1^	28.0 ± 4.6	34.5 ± 1.2	34.7 ± 1.3	35.1 ± 1.3
*n* (Ar)	1.15 ± 0.16	1.11 ± 0.13	1.017 ± 0.025	1.45 ± 0.12
*n* (He)	1.38 ± 0.27	1.31 ± 0.11	1.30 ± 0.12	1.32 ± 0.12
*B* (Ar)/K^–1^	n/a	n/a	n/a	(3.6 ± 1.0) × 10^–4^
*A*_CH+H_2__/cm^3^ s^–1^	(3.49 ± 0.47) × 10^–10^	(2.57 ± 0.24) × 10^–10^	(2.71 ± 0.27) × 10^–10^	(2.63 ± 0.26) × 10^–10^
*n*_CH+H_2__^∞^	–0.30 ± 0.44	0.15 ± 0.19	0.23 ± 0.20	0.22 ± 0.19
*A*_CH_2_+H_/cm^3^ s^–1^	(2.3 ± 4.4) × 10^–10^	(1.71 ± 0.11) × 10^–10^	(1.75 ± 0.11) × 10^–10^	(1.69 ± 0.11) × 10^–10^
*n*_CH_2_+H_^∞^	–0.05 ± 1.5	0.08 ± 0.11	0.03 ± 0.10	0.05 ± 0.10
Δ_r_*H*_1_(0 K)/kJ mol^–1^	14.9 ± 5.4	14.25^14^	14.25^14^	14.25^14^
χ^2^/degree of freedom	0.86	0.84	1.16	0.73

The energy transfer parameters for argon and helium are well-defined and are comparable with data from similar systems.^32^ As with other fits, ⟨Δ*E*⟩_d_, the average energy transferred in a downward direction, shows a positive temperature dependence, scaling in an almost linear fashion with temperature over the range 185–800 K. However, when high-temperature shock tube data are included, ⟨Δ*E*⟩_d_ shows a more complex temperature dependence as discussed below.

**Figure 5:**
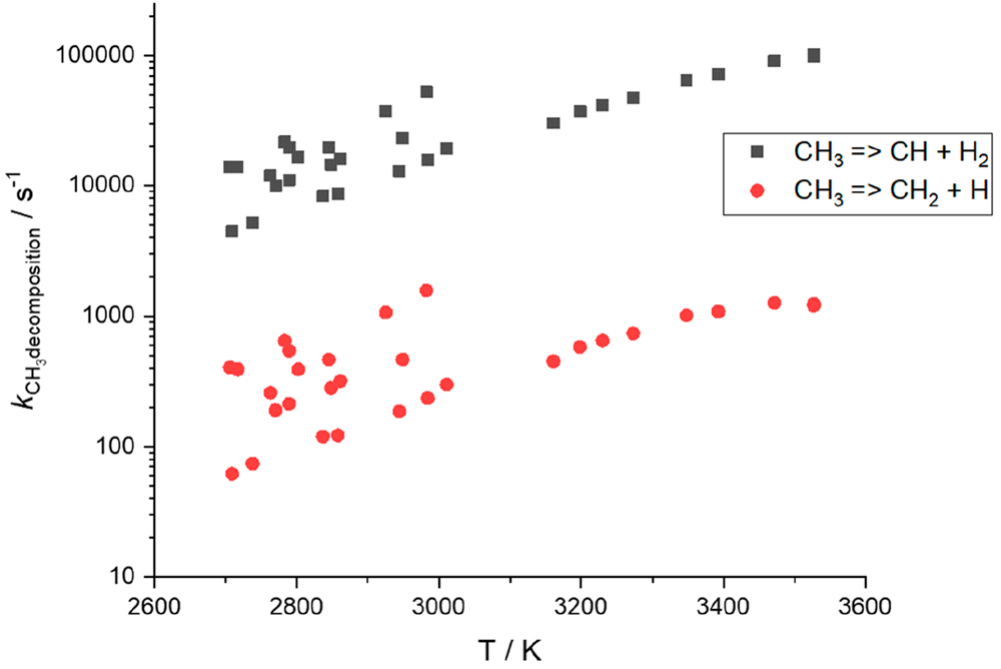
MESMER simulations of the rates of CH_3_ decomposition to CH + H_2_ (black ■) and ^3^CH_2_ + H (red ●) under the conditions used by Vasudevan et al.^40^ The reaction is dominated by production of CH and H_2_. From 2700–3000 K, multiple points at a given temperature are due to the variation in pressure from 0.7–4.2 bar.

We also included experimental data from Vasudevan et al.,^40^ who monitored the decomposition of CH_3_ via shock tube methods with direct detection of CH but also reported significant production of ^3^CH_2_ + H via direct observation of OH, which was considered as a proxy for ^3^CH_2_ + H. We are able to include the CH data in the MESMER analysis with good quality fits (Fit 3, Table 2). However, MESMER simulations show that production of the ^3^CH_2_ + H channel does not occur to any great extent under the experimental conditions of Vasudevan et al., as shown in Figure 5.

The lower-energy product channel of CH + H_2_ versus ^3^CH_2_ + H ensures that at the conditions of the shock tube experiments, any CH_3_* above threshold exits as CH + H_2_ rather than undergoing additional excitation to access the ^3^CH_2_ + H product channel. Vasudevan et al. suggested that CH and ^3^CH_2_ are formed in roughly equal amounts based on their observations of both CH and OH product. Their assignment of the yield assumed that the observed long-time OH signal could be solely assigned to the ^3^CH_2_ + H channel. However, it is known that CH + O_2_ as well as ^3^CH_2_ + O_2_ can lead to OH.^41,43,44^

Qualitatively similar arguments have been presented by Fulle and Hippler.^20^ At low pressures all of the activated CH_3_* will dissociate to CH and H_2_, but at the high pressure limit, while CH + H_2_ will be the dominant channel, approximately 25% of CH_3_ will dissociate to ^3^CH_2_ + H at ∼2000 K. However, the high-pressure limit will only be reached at pressures of several thousand bar, significantly greater than the pressures used by Vasudevan et al. (0.7–4.2 bar).

**Figure 6:**
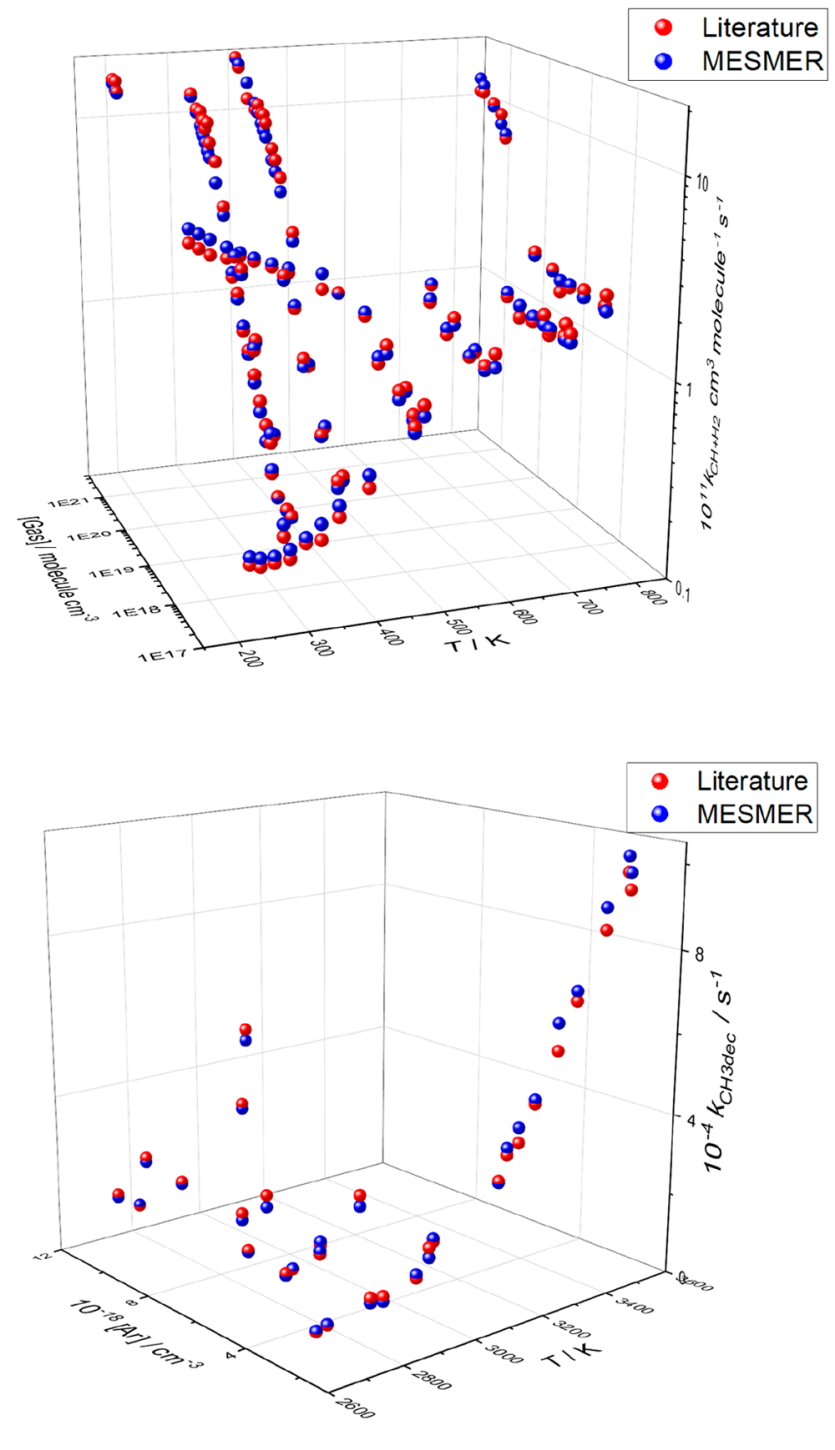
MESMER fit to literature data. The fitting parameters are given in the final column of Table 2. Upper panel: Fit to CH + H_2_ data. The low-pressure data at the front of the plot show the increase in *k*_1_ as a function of temperature; here the dominant channel is the formation of ^3^CH_2_ + H. The increase in *k*_1_ as a function of pressure can also be seen leading to increasing formation of CH_3_. Lower panel: Fit to CH_3_ → CH + H_2_ shock tube data. Again there is a strong increase with temperature and an increase with pressure, but the data are far from the high-pressure limit.

Figure 6 shows the fit to the datasets on the CH + H_2_ and CH_3_ decomposition reactions as a function of temperature and pressure. The literature data on CH + H_2_ cover the temperature and pressure ranges 185–800 K and 5–76000 Torr, and generally there is excellent agreement across the dataset between the experimental data and the MESMER fit. A similarly good fit is obtained when the direct measurements of CH from CH_3_ decomposition are included in Fit 3. The fit to the shock tube data is shown in the lower panel of Figure 6.

One further point to notice here is the function used to fit the energy transfer parameter ⟨Δ*E*⟩_d_from the complete set of experimental data. A simple *A* × (*T*/298 K)^*n*^ dependence is no longer suitable over this wider temperature range, and the energy transfer parameters at shock tube conditions are overestimated by this simple parametrization. Such effects have been noted in our previous studies on CH_3_ recombination.^38^ The parametrization of energy transfer by argon has been extended to include an additional temperature dependence to allow for the observed reduction in efficiency at high temperatures:
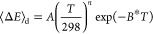
4Incorporating this additional parameter into the fit decreases the reduced χ^2^ parameter by approximately 40% from Fit 3 to Fit 4.

The final column of Table 2 shows our best estimates of the fitting parameters. The parameters for the limiting rate coefficients of the bimolecular reactions are well-defined, and the implications are discussed in the next subsection.

### Implications for *k*_–1_

**Figure 7:**
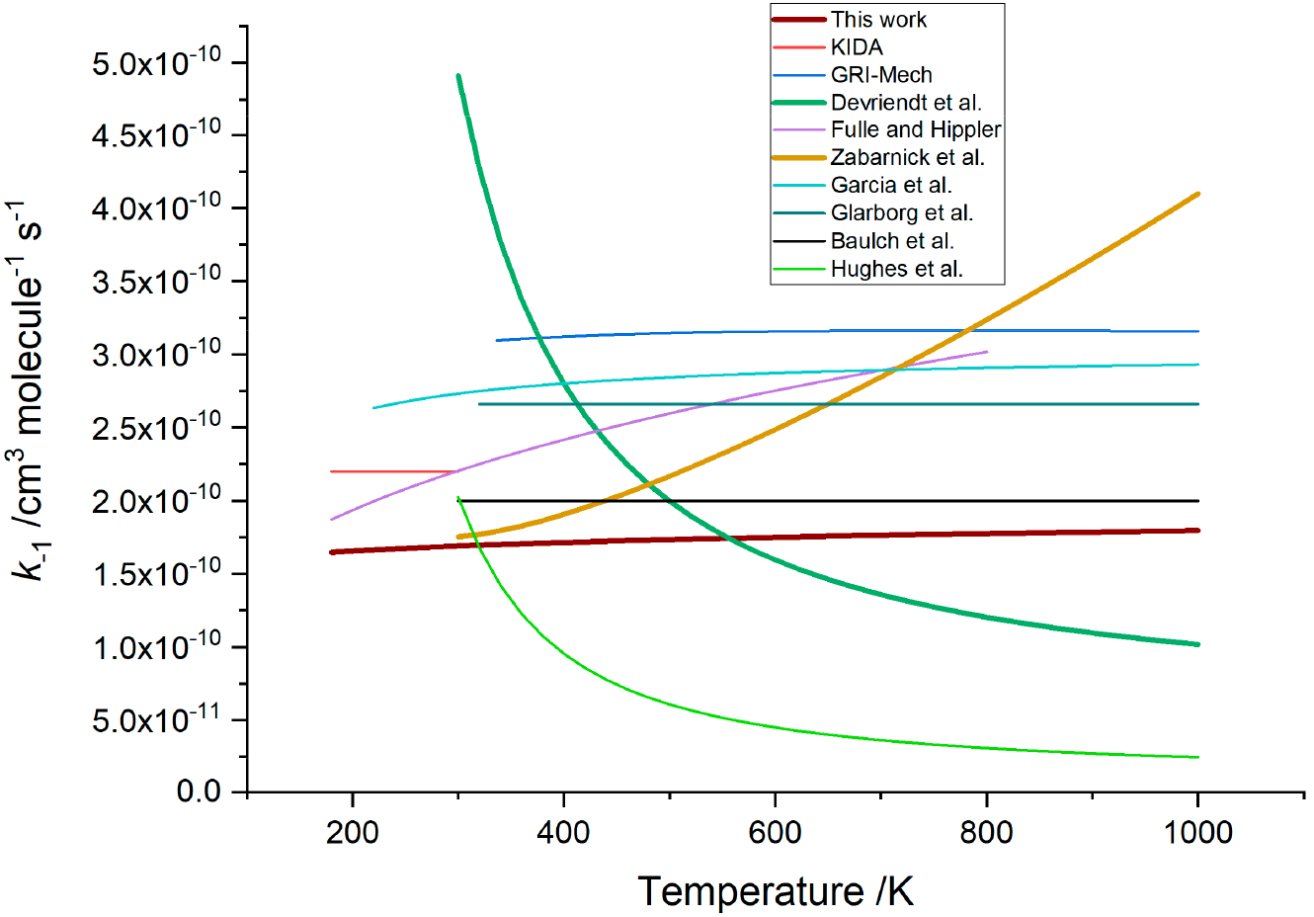
Temperature dependence of selected data on reaction −1, ^3^CH_2_ + H → CH + H_2_. Three traces have been highlighted to show the variation in temperature dependence. Devriendt et al. report a strong negative temperature dependence based on experimental studies where reaction −1 was monitored relative to ^3^CH_2_ + O. Zabarnick et al. report a positive temperature dependence based on experimental studies of reaction 1 and application of equilibrium. Finally, this work shows a temperature-independent value for *k*_–1_ using a similar approach to Zabarnick et al. but with updated thermochemistry.

The recommendation from this work is that the rate coefficient for the reaction ^3^CH_2_ + H → CH + H_2_ (reaction −1) can be parametrized as *k*_–1_(*T*) = (1.69 ± 0.11) × 10^–10^ × (*T*/298 K)^(0.05±0.010)^ cm^3^ molecule^–1^ s^–1^, where the errors are 1σ. Figure 7 shows the temperature dependence for *k*_–1_ based on both this work and various literature studies. This work, the theoretical calculations of Garcia et al.,^21^ and the recommendations of the Baulch et al.^45^ review are in excellent agreement on the lack of any significant temperature dependence, and room-temperature values vary over a relatively narrow range from (1.7 to ∼3) × 10^–10^ cm^3^ molecule^–1^ s^–1^. Our result is also in excellent agreement with the room-temperature study of Bohland et al.^26^ (1.83 × 10^–10^ cm^3^ molecule^–1^ s^–1^). This good level of agreement is in marked contrast to the conclusions of both the most recent direct study of Devriendt et al.^24^ and the earlier calculations based on measurements of *k*_1_ and application of equilibrium: *K*_1_ = *k*_1_/*k*_–1_ from Fulle and Hippler and Zabarnick et al. Given the importance of reaction −1 in converting relatively unreactive ^3^CH_2_ to reactive CH radicals, it is important for both combustion and planetary atmosphere modeling that *k*_–1_ is well-defined.

Direct studies of *k*_–1_ are challenging. Devriendt et al.^24^ generated ^3^CH_2_ from the reaction of O atoms with ketene, which efficiently produces ^3^CH_2_ but also forms other radicals. CH_2_ was monitored by mass spectrometry, but corrections had to be made for a component of the CH_2_ signal arising from ketene ionization. Finally, *k*_–1_ was not measured directly but was measured relative to the reaction of ^3^CH_2_ with O, and *k*_CH_2_+O_ was taken to be temperature-independent from a review of the relatively limited literature with a value of 1.3 × 10^–10^ cm^3^ molecule^–1^ s^–1^. Values close to room temperature, where a majority of the measurements of *k*_CH_2_+O_ have been made, are in reasonable agreement with this work. It may therefore be that the higher-temperature measurements of ^3^CH_2_ + O are in error. Devriendt et al. suggested that their observed negative temperature dependence is in good agreement with shock tube measurements (e.g., Frank et al.,^46^*k*_–1_ = 1.3 × 10^–11^ cm^3^ molecule^–1^ s^–1^) and rationalized the decrease in *k*_–1_ due to the more constrained transition state leading to CH + H_2_. However, a strong negative temperature dependence for *k*_–1_ is simply not consistent with the well-determined thermochemistry and kinetics for reaction 1, and other shock tube measurements give considerably higher values (e.g., Rohrig et al.,^23^ 2.34 × 10^–10^ cm^3^ molecule^–1^ s^–1^).

Zabarnick et al.^27^ and Fulle and Hippler^20^ used a similar analysis to this work to determine *k*_–1_ based on a calculation via the equilibrium constant. However, in such calculations, the value of the rate coefficient, in this case *k*_–1_, is extremely sensitive to the absolute thermochemistry of the reaction and its temperature dependence. In comparison to more recent studies, the temperature dependence of the thermochemistry used by Fulle and Hipplier and Zabarnick et al. was slightly weaker, and hence, the well-defined and significant positive activation energy for *k*_1_ translates to a weak positive temperature dependence for *k*_–1_.

The results of this work are in excellent agreement with the review of Baulch et al.,^45^ which is the basis of several combustion codes (e.g., Christensen and Konnov, 2017^47^). However, in general a wide range of values for *k*_–1_ have been used in combustion models. Hughes et al.^31^ considered in detail a number of reactions that influence methane chemistry, including reaction −1, and emphasized the important role that the ^3^CH_2_ + H reaction plays in generating reactive CH. The rate expression used, 1 × 10^–11^ exp(7500 J mol^–1^/*RT*) cm^3^ molecule^–1^ s^–1^, is in marked contrast to the recommendation of this work, in terms of both absolute value under combustion conditions and the temperature dependence: at 1000 K the rate coefficient is close to a factor 10 lower than the recommendation of this work. GRI-Mech 3.0^48^ uses a temperature-independent value for *k*_–1_ that is approximately a factor of 2 higher than that in this work. GRI-Mech is another common base mechanism used in more specialized combustion models. In contrast, while the Aramco mechanism^49^ also uses a temperature-independent value for *k*_–1_, the value of 5 × 10^–11^ cm^3^ molecule^–1^ s^–1^ for *k*_–1_ is approximately a factor of 3 less than that recommended in this work. Finally, the role of reaction −1 was also reviewed in the appendix of Glarborg et al.’s work on combustion mechanisms of nitrogen-containing species.^32^ Their recommended temperature-independent rate coefficient is *k*_–1_ = 2.66 × 10^–10^ cm^3^ molecule^–1^ s^–1^.

Reaction −1 is also important in planetary chemistry: at temperatures of 100–200 K relevant for the upper atmospheres of the outer planets, extrapolations of either the Devriendt et al. data (which would be beyond the temperature range of the experiments) or the Fulle and Hippler data would produce very different rate coefficients. However, many recent models will be based on the data evaluations of the KIDA group,^50^ and here the recommended rate coefficient from 10–298 K is a temperature-independent value of 2.2 × 10^–10^ cm^3^ molecule^–1^ s^–1^, which is only approximately 30% above the recommendation of this work. Therefore, the impact on chemical models based on KIDA data should not be too great. However, well-cited earlier modeling studies such as the work of Wilson and Atreya^51^ or Vuitton et al.^33,52^ on benzene formation in Titan use data on *k*_–1_ from Zabarnick et al. and Fulle and Hippler, respectively, and therefore, changes may be required to account for variations in reactive CH formation.

## Conclusions

New results on the CH + H_2_ reaction (reaction 1) have been obtained using laser flash photolysis combined with laser-induced detection of CH. Care needs to be taken to avoid interference from other species. In contrast to Brownsword et al.,^16^ no anomalous pressure dependence was observed. Our own data and other literature data on reaction 1 and the decomposition kinetics of methyl radicals have been combined and analyzed using a master equation code. The analysis provides a precise determination of the kinetics of the reaction ^3^CH_2_ + H → CH + H_2_ (reaction −1): *k*_–1_(*T*) = (1.69 ± 0.11) × 10^–10^ × (*T*/298 K)^(0.05±0.010)^ cm^3^ molecule^–1^ s^–1^. The magnitude and temperature dependence of *k*_–1_ differ significantly from both some experimental data and the values used in some combustion and planetary atmosphere models.

## Supporting Information Available

Experimental rate coefficients *k*_1_ as a function of temperature and pressure, detailed analysis of the three-state model proposed by Brownsword et al., and an example MESMER input file (PDF)
