# Supplementary material for: Studies on the Kinetics of the CH + H2 Reaction and Implications for the Reverse Reaction, 3CH2 + H
Source: J Phys Chem A. 2023 Mar 1;127(10):2367–75. doi: 10.1021/acs.jpca.2c08097 (PMC10026075; doi:10.1021/acs.jpca.2c08097)
Supplement: Supplementary file 1 — jp2c08097_si_001.pdf [file jp2c08097_si_001.pdf]

Studies on the Kinetics of the CH + H_2 Reaction and Implications for the Reverse Reaction, $^3CH_2$ + H

Mark A. Blitz[1,2*], Lavinia Onel[1], Struan H. Robertson[3] and Paul W. Seakins[1*]
1 – School of Chemistry, University of Leeds, Leeds, LS2 9JT, UK
2 – NCAS, University of Leeds, Leeds, LS2 9JT, UK
3 – Dassault Systemes, 334 Science Park, Cambridge, CB2 0WN, UK

Supplementary Information

This file contains a table of experimental data on the CH + H_2, further discussion of the three state model proposed by Brownsword et al.[1] and an example MESMER input file used to extract information on the CH + H_2 = $^3CH_2$ + H system.

1. Summary of Experimental Data

Table S1. Summary of Experimental Data

Temperature / K	Bath Gas, M	Pressure of M / Torr	[M] /molecule cm^3	k_1 /cm^3 molecule^{-1} s^{-1}
298	Ar	8.93	2.88×10^{17}	$(3.00 \pm 0.24^a\ [0.38]^b) \times 10^{-12}$
298	Ar	30.1	9.71×10^{17}	$(6.11 \pm 0.20\ [0.64]) \times 10^{-12}$
298	Ar	110.8	3.57×10^{18}	$(1.49 \pm 0.03\ [0.15]) \times 10^{-11}$
298	Ar	304.4	9.82×10^{18}	$(2.95 \pm 0.12\ [0.32]) \times 10^{-11}$
373	Ar	10.8	2.80×10^{17}	$(4.44 \pm 0.21\ [0.49]) \times 10^{-12}$
373	Ar	34.9	9.04×10^{17}	$(6.37 \pm 0.18\ [0.66]) \times 10^{-12}$
373	Ar	106.1	2.74×10^{18}	$(1.08 \pm 0.02\ [0.11]) \times 10^{-11}$
373	Ar	273.6	7.10×10^{18}	$(1.86 \pm 0.06\ [0.20]) \times 10^{-11}$
473	Ar	9.27	1.89×10^{17}	$(8.32 \pm 0.35\ [0.90]) \times 10^{-12}$
473	Ar	25.3	5.17×10^{17}	$(9.55 \pm 0.16\ [0.97]) \times 10^{-12}$
473	Ar	88.5	1.81×10^{18}	$(1.08 \pm 0.01\ [0.11]) \times 10^{-11}$
473	Ar	241.3	4.90×10^{18}	$(1.58 \pm 0.06\ [0.17]) \times 10^{-11}$
573	Ar	11.6	1.95×10^{17}	$(1.52 \pm 0.05\ [0.16]) \times 10^{-11}$
573	Ar	27.0	4.56×10^{17}	$(1.34 \pm 0.02\ [0.13]) \times 10^{-11}$
573	Ar	100.1	1.68×10^{18}	$(1.44 \pm 0.03\ [0.14]) \times 10^{-11}$
573	Ar	274.4	4.62×10^{18}	$(1.83 \pm 0.07\ [0.19]) \times 10^{-11}$
673	Ar	5.3	7.60×10^{16}	$(2.07 \pm 0.05\ [0.22]) \times 10^{-11}$
673	Ar	19.5	2.80×10^{17}	$(2.14 \pm 0.04\ [0.21]) \times 10^{-11}$
673	Ar	141.5	2.03×10^{18}	$(2.05 \pm 0.04\ [0.21]) \times 10^{-11}$
673	He	6.7	9.56×10^{16}	$(2.46 \pm 0.04\ [0.25]) \times 10^{-11}$
673	He	14.6	2.09×10^{17}	$(1.99 \pm 0.10\ [0.22]) \times 10^{-11}$
673	He	34.1	4.49×10^{17}	$(1.95 \pm 0.07\ [0.21]) \times 10^{-11}$
673	He	71.3	1.02×10^{18}	$(1.84 \pm 0.05\ [0.19]) \times 10^{-11}$
748	Ar	12.4	1.60×10^{17}	$(2.44 \pm 0.09\ [0.26]) \times 10^{-11}$
748	Ar	70.3	9.07×10^{17}	$(2.37 \pm 0.05\ [0.24]) \times 10^{-11}$
748	Ar	174.2	2.25×10^{18}	$(2.61 \pm 0.15\ [0.30]) \times 10^{-11}$
748	Ar	258.3	3.33×10^{18}	$(2.73 \pm 0.09\ [0.29]) \times 10^{-11}$

a – This error is the statistical error from the bimolecular fit at the 1σ level.
b – The error shown in square brackets is the combination of the statistical and estimated systematic error (10%) combined in quadrature.

2. Modelling of Negative Pressure Dependence Using the Three State Model Proposed by Brownsword et al.

Brownsword et al.[1] qualitatively explained their observed negative pressure dependence (a 37% reduction in the rate coefficient of CH + H_2 at 744 K from 4 to 100 Torr) via a three state model of the methyl intermediate, as shown in Figure S1. The highest energy state, CH_3^{**}, has sufficient energy to form $^3CH_2$ + H or re-dissociate back to CH + H_2 and can be collisionally stabilized to the intermediate state, CH_3^*. CH_3^*, which can only re-dissociate back to CH + H_2 or be collisionally stabilized to CH_3 and finally, CH_3 representing methyl radicals below the threshold to re-dissociate back to reagents. Brownsword et al. argued that at high temperatures, CH_3^{**} is preferentially populated; at very low pressures CH_3^{**} proceeds only forwards to products or back to reagents, but as pressure is increased, more CH_3^{**} is stabilized to CH_3^*, rather than proceeds to products. If a substantial proportion of CH_3^* re-dissociates, the observed rate coefficient for CH removal will actually decrease with pressure.

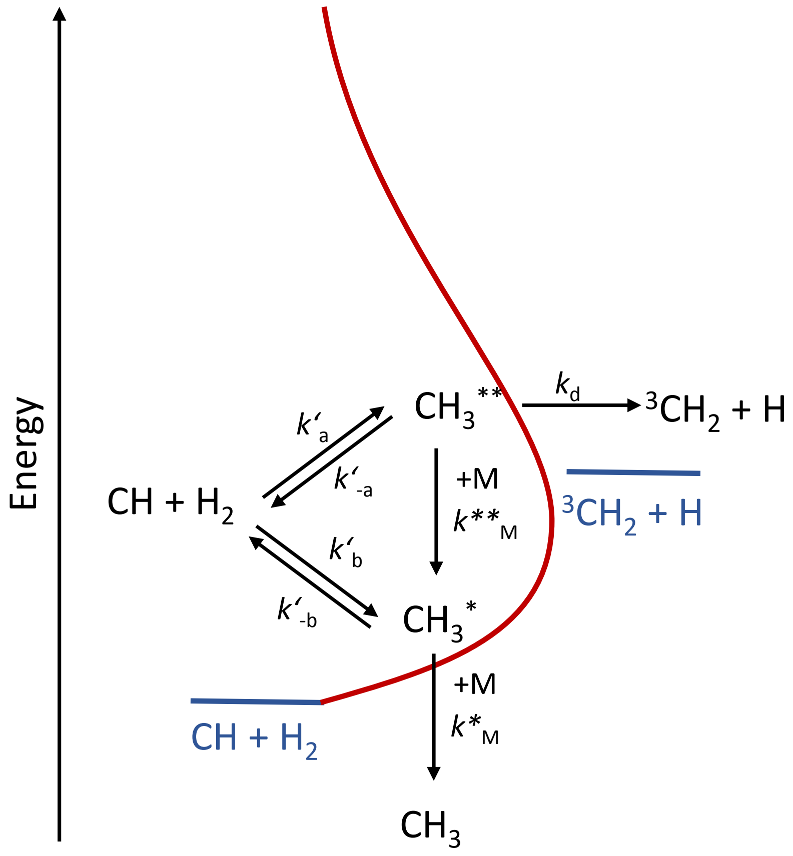

Figure S1: Schematic figure of the three state mechanism proposed by Brownsword et al. The blue lines show the energy thresholds for CH + H_2 and $^3CH_2$ + H. The red line represents the energy distribution of the initially formed CH_3 emphasising that, at high temperatures, a majority of the methyl complex is formed above the barrier to $^3CH_2$ + H, i.e. in the simplified model $[CH_3^{**}]_0 > [CH_3^*]_0$.

Figure S1 is loosely based on a figure from Brownsword et al. with the mechanism shown schematically. k'_a and k'_b are the pseudo-first-order rate coefficients for the formation of CH_3^{**} and CH_3^{*} respectively and k'_{-a} and k'_{-b} are the corresponding unimolecular decomposition reactions to reactants. k_d is the unimolecular rate coefficient for the formation of $^3CH_2$ + H from CH_3^{**} and k^{**}_M and k^{*}_M are the bimolecular rate coefficients for collisional relaxation of CH_3^{**} to CH_3^{*} and CH_3^{*} to stabilized CH_3 respectively.

Applying a steady-state treatment to CH_3^{**} and CH_3^{*} gives the following expression for the observed pseudo-first-order rate coefficient for CH removal in the presence of excess H_2:

$$k_{obs} = (k'_a + k'_b) - \frac{k_{-a}k'_a}{(k_d + k_{-a} + k^{**}_M[M])} - \frac{k_{-b}k'_b}{(k_{-b} + k^{*}_M[M])} - \frac{k_{-b}k'_a k^{**}_M[M]}{(k_d + k_{-a} + k^{**}_M[M])(k_{-b} + k^{*}_M[M])} \quad \text{ES1}$$

The first term $(k'_a + k'_b)$, is the total rate coefficient for production of excited CH_3. The second term, $\frac{k_{-a}k'_a}{(k_d + k_{-a} + k^{**}_M[M])}$, is the rate coefficient for the return of CH_3^{**} to reactants and the third term, $\frac{k_{-b}k'_b}{(k_{-b} + k^{*}_M[M])}$, is the equivalent term for CH_3^{*} dissociating back to reactants. The final term, $\frac{k_{-b}k'_a k^{**}_M[M]}{(k_d + k_{-a} + k^{**}_M[M])(k_{-b} + k^{*}_M[M])}$, is the rate coefficient for the production of CH + H_2 via collisional deactivation of CH_3^{**} to CH_3^{*} followed by re-dissociation back to reactants.

Figure S2 shows how these four terms vary with pressure and the typical behaviour of k_{obs}. The pressure dependent terms for reforming the reactants (terms 2 and 3) show a monotonic decrease with [M], but, particularly interesting is the behaviour of the fourth term, which rises from zero to reach a maximum before decreasing again at high pressure. At very low pressures, little CH_3^{**} is collisionally relaxed to CH_3^{*}, whereas at very high pressures, stabilization of CH_3^{*} to CH_3 out competes re-dissociation to reactants. It is this behaviour of the fourth term that gives rise to the potential for a negative pressure dependence; *if* the over a given increase in pressure, the increase in the fourth term outweighs the decreases in the second and third terms, then k_{obs} can decrease.

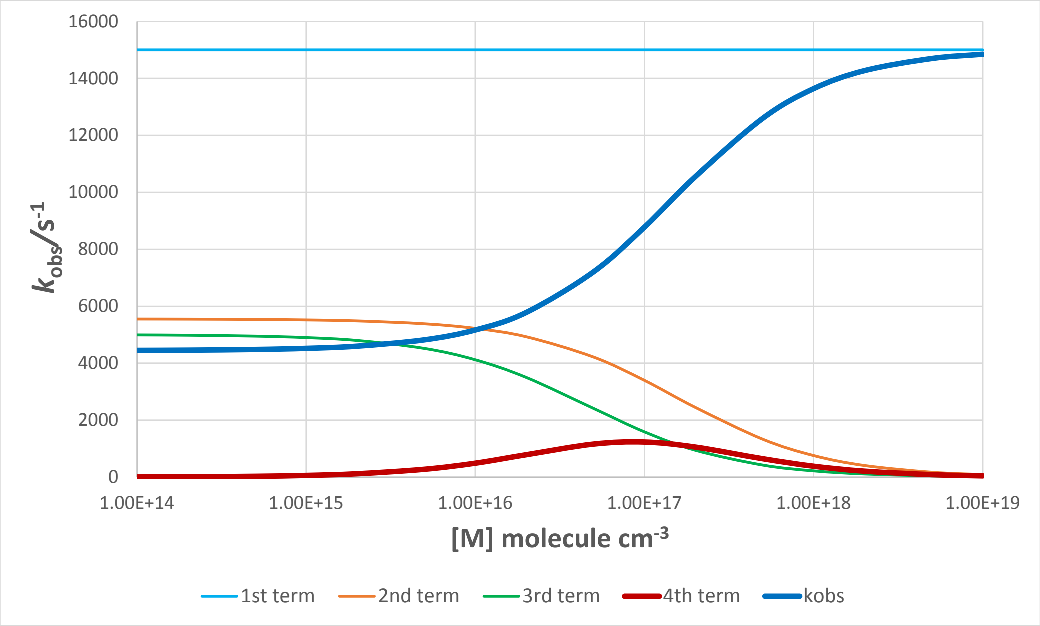

Figure S2: Typical 'S' shaped pressure dependence of k_{obs} and the pressure dependence of the four constituent terms. This simulation has a 2:1 ratio of $[CH_3^{**}]$:$[CH_3^{*}]$, an 4:3 ratio of k_M^{**}: k_M^{*} and a 5:2 ratio of k_{-a}: k_{-b}.

Figure S3a shows an example of a negative pressure dependence, but even for this fairly minimal decrease in k_{obs}, approximately 5%, compared to the ~33% decrease observed by Brownsword et al., the parameters required to generate this behaviour are at the bounds of realistic behaviour. Negative pressure dependence is promoted when:

1. $k^{**}_M > k^{*}_M$. At a given pressure population is more rapidly collisionally deactivated from CH_3^{**} to CH_3^{*} than from CH_3^{*} to CH_3, promoting the formation of CH_3^{*} and allowing for more re-dissociation from CH_3^{*} to reaction.

2. Relatively high values of k_{-b} compared to k_{-a}. However, rate coefficients for re-dissociation should increase rapidly with energy, so one would expect that $k'_{-a} >> k'_{-b}$

3. Relatively low initial population of CH_3^{*} compared to CH_3^{**}, so that more CH_3^{*} can be formed indirectly. At high temperatures, Brownsword et al. argued that CH_3** would be preferentially populated, but in reality (and as is shown in a master equation analysis), only those CH_3^{**} molecules that are formed relatively close to the $^3CH_2$ + H threshold can contribute to the negative pressure dependence. Those molecules formed at much higher energy are much more likely to react unimolecularly and hence require very high pressures for relaxation to complete; such high pressures then mean that virtually all of the CH_3^{*} that is formed indirectly, will be stabilized and hence not contribute to reactant regeneration.

S3a

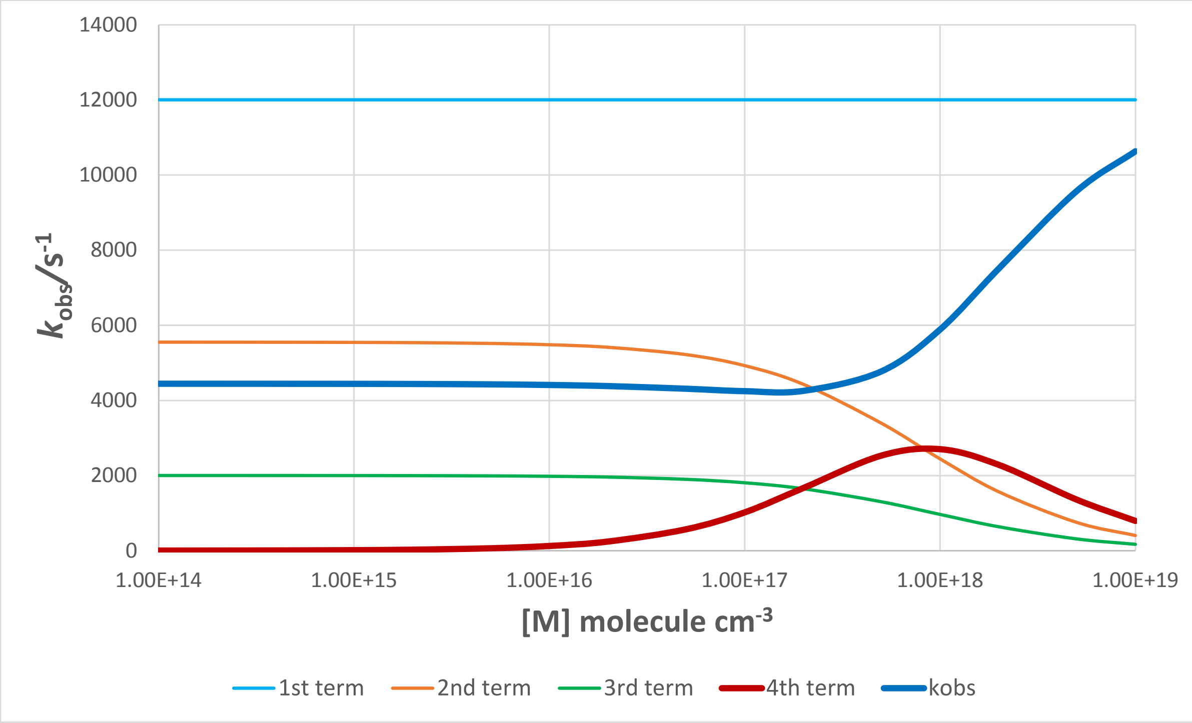

S3b

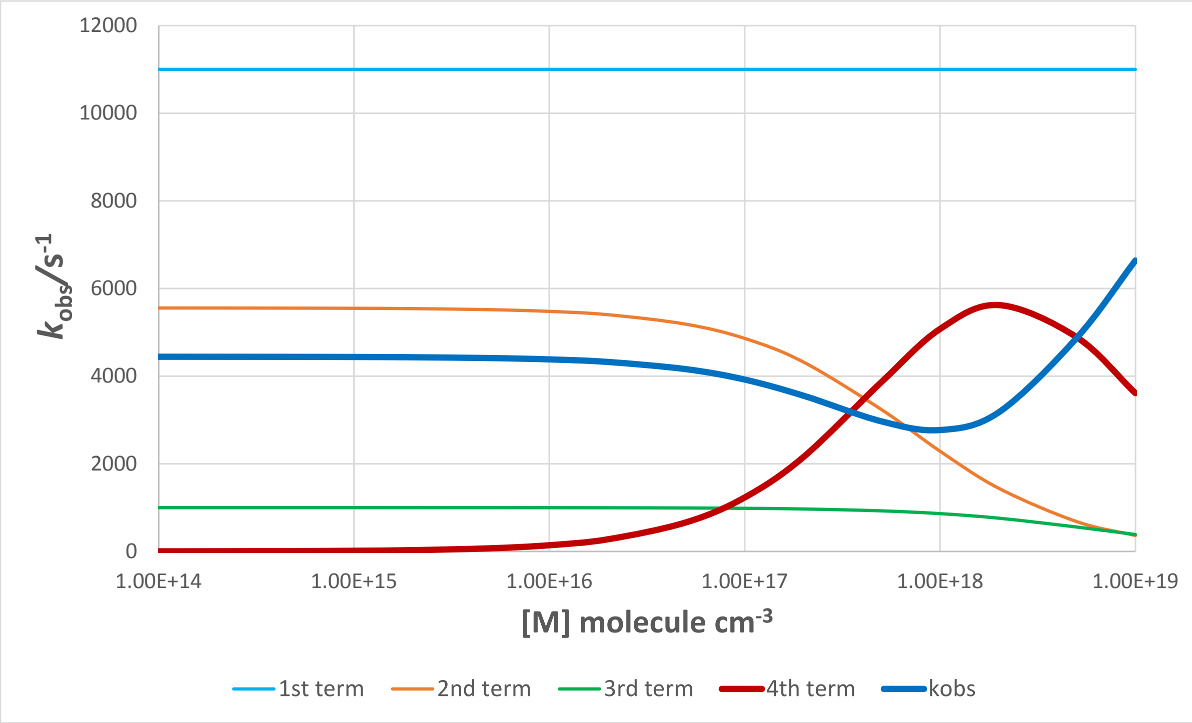

Figure S3: Simulations showing (a) minor negative dependence (decrease of ~4%) in k_{obs}. This requires a 5:1 ratio of [CH_3^{**}]:[CH_3^{*}], an 8:3 ratio of k_M^{**}: k_M^{*} and a 5:4 ratio of k_{-a}: k_{-b}. (b) to get the ~37% variation in k_{obs} to match the behaviour reported by Brownsword et al. requires a 10:1 ratio of [CH_3^{**}]:[CH_3^{*}], an 9:1 ratio of k_M^{**}: k_M^{*} and a 5:9 ratio of k_{-a}: k_{-b}.

Figure S3b shows an example where k_{obs} decreases by around 37%, but the values required (e.g. $k'_{-b} > k'_{-a}$) are clearly unrealistic.

In summary, we have used the three state CH_3 mechanism proposed by Brownsword et al. and shown that potentially it can qualitatively generate the negative pressure behaviour shown in the high temperature data. However, neither a full master equation, nor the simpler two state system can replicate the degree of negative pressure dependence observed by Brownsword et al. with realistic parameters.

3. Example MESMER Input file

```
<me:title>CH + H2</me:title>

 <moleculeList>
  <molecule id="CH">
   <propertyList>
    <property title="Energy" dictRef="me:ZPE">
     <scalar units="kJ/mol">592.825</scalar>
    </property>
    <property title="SpinMultiplicity" dictRef="me:spinMultiplicity">
     <scalar>2</scalar>
    </property>
    <property title="Vibrational Frequencies" dictRef="me:vibFreqs">
     <!-- <array units="cm-1">2947.89</array> -->
                  <!-- experimental -->
                    <array units="cm-1">2733</array>
    </property>
    <property title="Rotational Constants" dictRef="me:rotConsts">
     <!-- <array units="cm-1"> 14.619 </array> -->
                  <!-- experimental  -->
                   <array units="cm-1"> 14.45988 </array>
    </property>
    <property title="Symmetry Number" dictRef="me:symmetryNumber">
     <scalar>1</scalar>
    </property>
    <property dictRef="me:MW">
     <scalar units="amu">13</scalar>
    </property>

                  <!-- RMG-GRI-Mech -->
                   <property dictRef="me:epsilon">
     <scalar>56</scalar>
    </property>
    <property dictRef="me:sigma">
     <scalar>2.75</scalar>
    </property>

   </propertyList>
   <me:DOSCMethod xsi:type="QMRotors"/>
         <me:energyTransferModel xsi:type="me:ExponentialDown">
```

```
                        <me:deltaEDown bathGas="Ar" units="cm-1" lower="20" upper="400"
stepsize="10.0">54.812</me:deltaEDown>
        <me:deltaEDownTExponent bathGas="Ar" referenceTemperature="298" lower="0" upper="2"
stepsize="0.02" >1.45328</me:deltaEDownTExponent>
        <!-- <me:deltaEDownTActivation bathGas="Ar" units="K-1" lower="-1.0" upper="1.0" stepsize="1e-5" >-
0.000357883</me:deltaEDownTActivation> -->

        <me:deltaEDown bathGas="He" units="cm-1" lower="20" upper="400"
stepsize="10.0">35.0873</me:deltaEDown>
        <me:deltaEDownTExponent bathGas="He" referenceTemperature="298" lower="0" upper="2"
stepsize="0.02">1.31866</me:deltaEDownTExponent>

      </me:energyTransferModel>
    </molecule>


    <molecule id="H2">
      <propertyList>
        <property title="Energy" dictRef="me:ZPE">
          <scalar units="kJ/mol">0</scalar>
        </property>
        <property title="Vibrational Frequencies" dictRef="me:vibFreqs">
                        <array units="cm-1"> 4161</array>
          <!-- <array units="cm-1"> 4516.40 </array> -->
        </property>
        <property title="Rotational Constants" dictRef="me:rotConsts">
          <!-- <array units="cm-1">61.459 </array> -->
                        <!-- experimental -->
                          <array units="cm-1">60.853</array>
        </property>
        <property title="Symmetry Number" dictRef="me:symmetryNumber">
          <scalar>2</scalar>
        </property>
        <property dictRef="me:MW">
          <scalar units="amu">2</scalar>
        </property>
                        <!-- RMG-GRI-Mech -->
                        <property dictRef="me:epsilon">
          <scalar>26</scalar>
        </property>
        <property dictRef="me:sigma">
          <scalar>2.92</scalar>
        </property>
      </propertyList>
      <me:DOSCMethod xsi:type="QMRotors"/>
                <me:energyTransferModel xsi:type="me:ExponentialDown">


                <me:deltaEDown bathGas="Ar" units="cm-1" lower="20" upper="400"
stepsize="10.0">54.812</me:deltaEDown>
        <me:deltaEDownTExponent bathGas="Ar" referenceTemperature="298" lower="0" upper="2"
stepsize="0.02" >1.45328</me:deltaEDownTExponent>
        <!-- <me:deltaEDownTActivation bathGas="Ar" units="K-1" lower="-1.0" upper="1.0" stepsize="1e-5" >-
0.000357883</me:deltaEDownTActivation> -->

        <me:deltaEDown bathGas="He" units="cm-1" lower="20" upper="400"
stepsize="10.0">35.0873</me:deltaEDown>
```

```
        <me:deltaEDownTExponent bathGas="He" referenceTemperature="298" lower="0" upper="2"
stepsize="0.02">1.31866</me:deltaEDownTExponent>

      </me:energyTransferModel>
    </molecule>


    <molecule id="CH3">
      <propertyList>
        <property title="Energy" dictRef="me:ZPE">


          <!-- <scalar units="kJ/mol">-433.163</scalar> -->
                         <!-- Active Thermo -->
                           <scalar units="kJ/mol">149.866</scalar>
        </property>
        <property title="SpinMultiplicity" dictRef="me:spinMultiplicity">
          <scalar>2</scalar>
        </property>
        <property title="Vibrational Frequencies" dictRef="me:vibFreqs">
                        <!-- experimental -->
                         <array units="cm-1">3004 606 3161 3161 1396 1396</array>

        </property>
        <property title="Rotational Constants" dictRef="me:rotConsts">
          <array units="cm-1"> 9.649 9.649 4.824 </array>
        </property>
        <property title="Symmetry Number" dictRef="me:symmetryNumber">
          <scalar>6</scalar>
        </property>
        <property dictRef="me:MW">
          <scalar units="amu">15</scalar>
        </property>
                         <property dictRef="me:epsilon">
                         <!-- RMG-GRI-Mech -->
          <scalar>100</scalar>
        </property>
        <property dictRef="me:sigma">
          <scalar>3.8</scalar>
        </property>
      </propertyList>
      <me:DOSCMethod xsi:type="QMRotors"/>

       <me:energyTransferModel xsi:type="me:ExponentialDown">

    <me:deltaEDown bathGas="Ar" units="cm-1" lower="20" upper="400"
stepsize="10.0">54.812</me:deltaEDown>
        <me:deltaEDownTExponent bathGas="Ar" referenceTemperature="298" lower="0" upper="2"
stepsize="0.02" >1.45328</me:deltaEDownTExponent>
        <!-- <me:deltaEDownTActivation bathGas="Ar" units="K-1" lower="-1.0" upper="1.0" stepsize="1e-5" >-
0.000357883</me:deltaEDownTActivation> -->


        <me:deltaEDown bathGas="He" units="cm-1" lower="20" upper="400"
stepsize="10.0">35.0873</me:deltaEDown>
        <me:deltaEDownTExponent bathGas="He" referenceTemperature="298" lower="0" upper="2"
stepsize="0.02">1.31866</me:deltaEDownTExponent>
```

```
    </me:energyTransferModel>

   <me:reservoirSize units="kJ/mol">-20.0</me:reservoirSize>
   </molecule>

   <molecule id="CH2">
    <propertyList>
     <property title="Energy" dictRef="me:ZPE">
                    <scalar units="kJ/mol" lower="380" upper="410" stepsize="0.01">391.06</scalar>
      <!-- <scalar units="kJ/mol">391.06</scalar> -->
     </property>
     <property title="SpinMultiplicity" dictRef="me:spinMultiplicity">
      <scalar>3</scalar>
     </property>
     <property title="Vibrational Frequencies" dictRef="me:vibFreqs">
      <array units="cm-1"> 1131.92 3199.87 3432.23 </array>
     </property>
     <property title="Rotational Constants" dictRef="me:rotConsts">
      <array units="cm-1"> 53.052 8.620 7.415 </array>
     </property>
     <property title="Symmetry Number" dictRef="me:symmetryNumber">
      <scalar>2</scalar>
     </property>
     <property dictRef="me:MW">
      <scalar units="amu">14</scalar>
     </property>
                    <!-- RMG-GRI-Mech -->
                     <property dictRef="me:epsilon">
      <scalar>100</scalar>
     </property>
     <property dictRef="me:sigma">
      <scalar>3.8</scalar>
     </property>


    </propertyList>
    <me:DOSCMethod xsi:type="QMRotors"/>
           <me:energyTransferModel xsi:type="me:ExponentialDown">

                    <me:deltaEDown bathGas="Ar" units="cm-1" lower="20" upper="400"
stepsize="10.0">54.812</me:deltaEDown>
     <me:deltaEDownTExponent bathGas="Ar" referenceTemperature="298" lower="0" upper="2"
stepsize="0.02" >1.45328</me:deltaEDownTExponent>
     <!-- <me:deltaEDownTActivation bathGas="Ar" units="K-1" lower="-1.0" upper="1.0" stepsize="1e-5" >-
0.000357883</me:deltaEDownTActivation> -->

     <me:deltaEDown bathGas="He" units="cm-1" lower="20" upper="400"
stepsize="10.0">35.0873</me:deltaEDown>
     <me:deltaEDownTExponent bathGas="He" referenceTemperature="298" lower="0" upper="2"
stepsize="0.02">1.31866</me:deltaEDownTExponent>

    </me:energyTransferModel>
   </molecule>


   <molecule id="H">
```

```
      <propertyList>
        <property title="Energy" dictRef="me:ZPE">
          <scalar units="kJ/mol">216.034</scalar>
        </property>
        <property title="SpinMultiplicity" dictRef="me:spinMultiplicity">
          <scalar>2</scalar>
        </property>
        <property dictRef="me:MW">
          <scalar units="amu">1</scalar>
        </property>
                        <!-- RMG-GRI-Mech -->
                          <property dictRef="me:epsilon">
          <scalar>101</scalar>
        </property>
        <property dictRef="me:sigma">
          <scalar>2.05</scalar>
        </property>

      </propertyList>
              <me:energyTransferModel xsi:type="me:ExponentialDown">

                        <me:deltaEDown bathGas="Ar" units="cm-1" lower="20" upper="400"
stepsize="10.0">54.812</me:deltaEDown>
        <me:deltaEDownTExponent bathGas="Ar" referenceTemperature="298" lower="0" upper="2"
stepsize="0.02" >1.45328</me:deltaEDownTExponent>
        <!-- <me:deltaEDownTActivation bathGas="Ar" units="K-1" lower="-1.0" upper="1.0" stepsize="1e-5" >-
0.000357883</me:deltaEDownTActivation> -->


        <me:deltaEDown bathGas="He" units="cm-1" lower="20" upper="400"
stepsize="10.0">35.0873</me:deltaEDown>
        <me:deltaEDownTExponent bathGas="He" referenceTemperature="298" lower="0" upper="2"
stepsize="0.02">1.31866</me:deltaEDownTExponent>


      </me:energyTransferModel>
    </molecule>


            <molecule id="N2" description="nitrogen">
        <atomArray>
          <atom id="a1" elementType="N" />
          <atom id="a2" elementType="N" />
        </atomArray>
        <bondArray>
          <bond atomRefs2="a2 a1" order="3" />
        </bondArray>
        <propertyList>
          <property dictRef="me:epsilon">
            <scalar>48</scalar>
          </property>
          <property dictRef="me:sigma">
            <scalar>3.9</scalar>
          </property>
          <property dictRef="me:MW">
            <scalar units="amu">28.0</scalar>
          </property>
```

```
    </propertyList>
  </molecule>

      <molecule id="Ar" description="argon">
          <atomArray>
              <atom elementType="Ar"/>
          </atomArray>
          <propertyList>
              <property dictRef="me:epsilon">
                  <scalar>114</scalar>
              </property>
              <property dictRef="me:sigma">
                  <scalar>3.47</scalar>
              </property>
              <property dictRef="me:MW">
                  <scalar units="amu">40</scalar>
              </property>
          </propertyList>
      </molecule>


  <molecule id="He">
    <propertyList>
      <property dictRef="me:epsilon">
        <scalar>10.2</scalar>
      </property>
      <property dictRef="me:sigma">
        <scalar>2.55</scalar>
      </property>
      <property dictRef="me:MW">
        <scalar units="amu">4.0</scalar>
      </property>
    </propertyList>
  </molecule>

</moleculeList>

<reactionList>


  <reaction id="RCHH2">
    <reactantList>
      <reactant>
        <molecule ref="CH" role="deficientReactant"/>
      </reactant>
      <reactant>
        <molecule ref="H2" role="excessReactant"/>
      </reactant>
    </reactantList>
    <productList>
      <product>
        <molecule ref="CH3" role="modelled"/>
      </product>
    </productList>
    <me:MCRCMethod xsi:type="me:MesmerILT">
      <!-- <me:preExponential>3.31e-10</me:preExponential> -->
```

```
      <me:preExponential units="cm3molecule-1s-1" lower="1.0E-11" upper="5E-10" stepsize="1.5E-
13">2.63E-10</me:preExponential>
      <me:activationEnergy units="kJ/mol">0.0</me:activationEnergy>
      <me:nInfinity lower="-1.4" upper="1.4" stepsize="0.005">0.226482</me:nInfinity>
      <!-- <me:nInfinity>-0.12</me:nInfinity> -->
    </me:MCRCMethod>
    <me:excessReactantConc>1.0E15</me:excessReactantConc>
  </reaction>

  <reaction id="RCH3">
    <reactantList>
      <reactant>
        <molecule ref="CH3" role="modelled"/>
      </reactant>
    </reactantList>
    <productList>
      <product>
        <molecule ref="CH2" role="sink"/>
      </product>
      <product>
        <molecule ref="H" role="sink"/>
      </product>
    </productList>
    <me:MCRCMethod xsi:type="me:MesmerILT">
      <!-- <me:preExponential units="cm3molecule-1s-1">2.2E-10</me:preExponential> -->
                         <me:preExponential units="cm3molecule-1s-1" lower="1.0E-11" upper="15E-10"
stepsize="1.5E-13">1.69E-10</me:preExponential>
      <me:activationEnergy units="kJ/mol" reverse="true">0.0</me:activationEnergy>
      <me:nInfinity lower="-1.4" upper="1.4" stepsize="0.005">0.0511287</me:nInfinity>
      <!-- <me:nInfinity>0.032</me:nInfinity> -->
    </me:MCRCMethod>
  </reaction>

 </reactionList>

 <me:conditions>
   <me:bathGas>Ar</me:bathGas>

 <me:PTs>

 <!--Fulle and Hippler 1997-->

<me:PTpair units="PPCC" P="6.4E20" T= "185" precision="qd"><me:bathGas> He </me:bathGas><me:experimentalRate ref1="CH" ref2="CH" error="3.0E4 ">16.0E4 </me:experimentalRate></me:PTpair>
<me:PTpair units="PPCC" P="7.1E20" T= "185" precision="qd"><me:bathGas> He </me:bathGas><me:experimentalRate ref1="CH" ref2="CH" error=" 3.0E4 ">  18.0E4 </me:experimentalRate></me:PTpair>
<me:PTpair units="PPCC" P="9.3E20" T= "185" precision="qd"><me:bathGas> He </me:bathGas><me:experimentalRate ref1="CH" ref2="CH" error=" 3.0E4 ">  18.0E4 </me:experimentalRate></me:PTpair>


<me:PTpair units="PPCC" P="4.3E19" T= "300" precision="qd"><me:bathGas> He </me:bathGas><me:experimentalRate ref1="CH" ref2="CH" error="0.8E4 ">   5.1E4 </me:experimentalRate></me:PTpair>
<me:PTpair units="PPCC" P="7.8E19" T= "300" precision="qd"><me:bathGas> He </me:bathGas><me:experimentalRate ref1="CH" ref2="CH" error=" 1.2E4 ">  8.1E4 </me:experimentalRate></me:PTpair>
<me:PTpair units="PPCC" P="1.3E20" T= "300" precision="qd"><me:bathGas> He </me:bathGas><me:experimentalRate ref1="CH" ref2="CH" error=" 1.3E4 ">  9.6E4 </me:experimentalRate></me:PTpair>
<me:PTpair units="PPCC" P="1.5E20" T= "300" precision="qd"><me:bathGas> He </me:bathGas><me:experimentalRate ref1="CH" ref2="CH" error=" 2.0E4 ">  12E4 </me:experimentalRate></me:PTpair>
<me:PTpair units="PPCC" P="1.8E20" T= "300" precision="qd"><me:bathGas> He </me:bathGas><me:experimentalRate ref1="CH" ref2="CH" error=" 2.0E4 ">  11E4 </me:experimentalRate></me:PTpair>
<me:PTpair units="PPCC" P="2.1E20" T= "300" precision="qd"><me:bathGas> He </me:bathGas><me:experimentalRate ref1="CH" ref2="CH" error=" 2.0E4 ">  12E4 </me:experimentalRate></me:PTpair>
<me:PTpair units="PPCC" P="2.3E20" T= "300" precision="qd"><me:bathGas> He </me:bathGas><me:experimentalRate ref1="CH" ref2="CH" error=" 2.0E4 ">  12E4 </me:experimentalRate></me:PTpair>
<me:PTpair units="PPCC" P="2.7E20" T= "300" precision="qd"><me:bathGas> He </me:bathGas><me:experimentalRate ref1="CH" ref2="CH" error=" 3.0E4 ">  13E4 </me:experimentalRate></me:PTpair>
<me:PTpair units="PPCC" P="3.8E20" T= "300" precision="qd"><me:bathGas> He </me:bathGas><me:experimentalRate ref1="CH" ref2="CH" error=" 2.0E4 ">  13E4 </me:experimentalRate></me:PTpair>
<me:PTpair units="PPCC" P="6.0E20" T= "300" precision="qd"><me:bathGas> He </me:bathGas><me:experimentalRate ref1="CH" ref2="CH" error=" 2.0E4 ">  15E4 </me:experimentalRate></me:PTpair>


 <me:PTpair units="PPCC" P="3.0E19" T= "400" precision="dd"><me:bathGas> He </me:bathGas><me:experimentalRate ref1="CH" ref2="CH" error="0.6E4 ">  3.7E4 </me:experimentalRate></me:PTpair>
<me:PTpair units="PPCC" P="7.4E19" T= "400" precision="dd"><me:bathGas> He </me:bathGas><me:experimentalRate ref1="CH" ref2="CH" error=" 1.2E4 ">  6.4E4 </me:experimentalRate></me:PTpair>
<me:PTpair units="PPCC" P="1.1E20" T= "400" precision="dd"><me:bathGas> He </me:bathGas><me:experimentalRate ref1="CH" ref2="CH" error=" 1.3E4 ">  7.6E4 </me:experimentalRate></me:PTpair>
<me:PTpair units="PPCC" P="1.4E20" T= "400" precision="dd"><me:bathGas> He </me:bathGas><me:experimentalRate ref1="CH" ref2="CH" error=" 1.4E4 ">  8.5E4 </me:experimentalRate></me:PTpair>
<me:PTpair units="PPCC" P="2.3E20" T= "400" precision="dd"><me:bathGas> He </me:bathGas><me:experimentalRate ref1="CH" ref2="CH" error=" 2.0E4 ">  11E4 </me:experimentalRate></me:PTpair>


<me:PTpair units="PPCC" P="2.7E20" T= "400" precision="dd"><me:bathGas> He </me:bathGas><me:experimentalRate ref1="CH" ref2="CH" error=" 2.0E4 ">  12E4 </me:experimentalRate></me:PTpair>
<me:PTpair units="PPCC" P="3.2E20" T= "400" precision="dd"><me:bathGas> He </me:bathGas><me:experimentalRate ref1="CH" ref2="CH" error=" 2.0E4 ">  12E4 </me:experimentalRate></me:PTpair>
<me:PTpair units="PPCC" P="4.2E20" T= "400" precision="dd"><me:bathGas> He </me:bathGas><me:experimentalRate ref1="CH" ref2="CH" error=" 3.0E4 ">  13E4 </me:experimentalRate></me:PTpair>
<me:PTpair units="PPCC" P="5.4E20" T= "400" precision="dd"><me:bathGas> He </me:bathGas><me:experimentalRate ref1="CH" ref2="CH" error=" 3.0E4 ">  12E4 </me:experimentalRate></me:PTpair>


<me:PTpair units="PPCC" P="2.0E21" T= "400" precision="dd"><me:bathGas> He </me:bathGas><me:experimentalRate ref1="CH" ref2="CH" error=" 4.0E4 ">  18E4 </me:experimentalRate></me:PTpair>
```

<me:PTpair units="PPCC" P="2.6E21" T= "400" precision="dd"><me:bathGas> He </me:bathGas><me:experimentalRate ref1="CH" ref2="CH" error=" 4.0E4 "> 20E4 </me:experimentalRate></me:PTpair>

<me:PTpair units="PPCC" P="2.2E20" T= "800" precision="d"> <me:bathGas>He</me:bathGas> <me:experimentalRate ref1="CH" ref2="CH" error=" 1.2E4 "> 7.6E4 </me:experimentalRate> </me:PTpair>
<me:PTpair units="PPCC" P="2.9E20" T= "800" precision="d"> <me:bathGas>He</me:bathGas> <me:experimentalRate ref1="CH" ref2="CH" error=" 2.0E4 "> 10.0E4 </me:experimentalRate> </me:PTpair>
<me:PTpair units="PPCC" P="4.7E20" T= "800" precision="d"> <me:bathGas>He</me:bathGas> <me:experimentalRate ref1="CH" ref2="CH" error=" 2.0E4 "> 11.0E4 </me:experimentalRate> </me:PTpair>
<me:PTpair units="PPCC" P="8.8E20" T= "800" precision="d"> <me:bathGas>He</me:bathGas> <me:experimentalRate ref1="CH" ref2="CH" error=" 2.0E4 "> 12E4 </me:experimentalRate> </me:PTpair>
<me:PTpair units="PPCC" P="11.0E20" T= "800" precision="d"> <me:bathGas>He</me:bathGas> <me:experimentalRate ref1="CH" ref2="CH" error=" 2.0E4 "> 12E4 </me:experimentalRate> </me:PTpair>

<!--McIlroy_Tully_1993-->

<me:PTpair units="Torr" P="8.2" T= "294" precision="qd"><me:bathGas> He </me:bathGas><me:experimentalRate ref1="CH" ref2="CH" error="1.89E2">1.89E3</me:experimentalRate></me:PTpair>
<me:PTpair units="Torr" P="12.5" T= "294" precision="qd"><me:bathGas> He </me:bathGas><me:experimentalRate ref1="CH" ref2="CH” error="2.25E2"> 2.25E3</me:experimentalRate></me:PTpair>
<me:PTpair units="Torr" P="19.0" T= "294" precision="qd"><me:bathGas> He </me:bathGas><me:experimentalRate ref1="CH" ref2="CH” error="3.272">3.27E3 </me:experimentalRate></me:PTpair>
<me:PTpair units="Torr" P="28.5" T= "294" precision="qd"><me:bathGas> He </me:bathGas><me:experimentalRate ref1="CH" ref2="CH” error="4.0E2"> 4.0E3</me:experimentalRate></me:PTpair>
<me:PTpair units="Torr" P="43" T= "294" precision="qd"><me:bathGas> He </me:bathGas><me:experimentalRate ref1="CH" ref2="CH” error="6.49E2">6.49E3</me:experimentalRate></me:PTpair>
<me:PTpair units="Torr" P="65" T= "294" precision="qd"><me:bathGas> He </me:bathGas><me:experimentalRate ref1="CH" ref2="CH” error=“7.99E2”>7.99E3</me:experimentalRate></me:PTpair>
<me:PTpair units="Torr" P="98" T= "294" precision="qd"><me:bathGas> He </me:bathGas><me:experimentalRate ref1="CH" ref2="CH” error=“10.2E2”>10.2E3</me:experimentalRate></me:PTpair>
<me:PTpair units="Torr" P="148" T= "294" precision="qd"><me:bathGas> He </me:bathGas><me:experimentalRate ref1="CH" ref2="CH” error=“12.8E2”>12.8E3</me:experimentalRate></me:PTpair>
<me:PTpair units="Torr" P="222" T= "294" precision="qd"><me:bathGas> He </me:bathGas><me:experimentalRate ref1="CH" ref2="CH” error=“15.1E2”>15.1E3</me:experimentalRate></me:PTpair>
<me:PTpair units="Torr" P="333" T= "294" precision="qd"><me:bathGas> He </me:bathGas><me:experimentalRate ref1="CH" ref2="CH” error=“22.1E2”>22.1E3</me:experimentalRate></me:PTpair>

<me:PTpair units="Torr" P="500" T= "294" precision="qd"><me:bathGas> He </me:bathGas><me:experimentalRate ref1="CH" ref2="CH”error=“25.3E2”>25.3E3</me:experimentalRate></me:PTpair>
<me:PTpair units="Torr" P="750" T= "294" precision="qd"><me:bathGas> He </me:bathGas><me:experimentalRate ref1="CH" ref2="CH”error=“30.1E2”>30.1E3</me:experimentalRate></me:PTpair>

<me:PTpair units="Torr" P="750" T= "313" precision="dd"><me:bathGas> He </me:bathGas><me:experimentalRate ref1="CH" ref2="CH”error=“30.6E2”>30.6E3</me:experimentalRate></me:PTpair>
<me:PTpair units="Torr" P="750" T= "333" precision="dd"><me:bathGas> He </me:bathGas><me:experimentalRate ref1="CH" ref2="CH”error=“28.8E2”>28.8E3</me:experimentalRate></me:PTpair>
<me:PTpair units="Torr" P="750" T= "359" precision="dd"><me:bathGas> He </me:bathGas><me:experimentalRate ref1="CH" ref2="CH”error=“26.6E2”>26.6E3</me:experimentalRate></me:PTpair>
<me:PTpair units="Torr" P="750" T= "383" precision="dd"><me:bathGas> He </me:bathGas><me:experimentalRate ref1="CH" ref2="CH”error=“24.6E2”>24.6E3</me:experimentalRate></me:PTpair>

<me:PTpair units="Torr" P="8.2" T= "316" precision="dd"><me:bathGas> He </me:bathGas><me:experimentalRate ref1="CH" ref2="CH”error=“2.24E2”>2.24E3</me:experimentalRate></me:PTpair>
<me:PTpair units="Torr" P="8.2" T= "337" precision="dd"><me:bathGas> He </me:bathGas><me:experimentalRate ref1="CH" ref2="CH”error=“2.3E2”>2.3E3</me:experimentalRate></me:PTpair>
<me:PTpair units="Torr" P="8.2" T= "362" precision="dd"><me:bathGas> He </me:bathGas><me:experimentalRate ref1="CH" ref2="CH”error=“2.95E2”>2.95E3</me:experimentalRate></me:PTpair>

<me:PTpair units="Torr" P="8.2" T= "403" precision="dd"><me:bathGas> He </me:bathGas><me:experimentalRate ref1="CH" ref2="CH”error=“4.026E2”>4.02E3</me:experimentalRate></me:PTpair>
<me:PTpair units="Torr" P="8.2" T= "466" precision="dd"><me:bathGas> He</me:bathGas><me:experimentalRate ref1="CH" ref2="CH”error=“7.72E2”>7.72E3</me:experimentalRate></me:PTpair>

<me:PTpair units="Torr" P="750" T= "240" precision="qd"><me:bathGas> He </me:bathGas><me:experimentalRate ref1="CH" ref2="CH”error=“36.1E2”>36.1E3</me:experimentalRate></me:PTpair>
<me:PTpair units="Torr" P="750" T= "254" precision="qd"><me:bathGas> He </me:bathGas><me:experimentalRate ref1="CH" ref2="CH”error=“33.8E2”>33.8E3</me:experimentalRate></me:PTpair>
<me:PTpair units="Torr" P="750" T= "270" precision="qd"><me:bathGas> He </me:bathGas><me:experimentalRate ref1="CH" ref2="CH”error=“31.3E2”>31.3E3</me:experimentalRate></me:PTpair>

<me:PTpair units="Torr" P="750" T= "433" precision="dd"><me:bathGas> He</me:bathGas><me:experimentalRate ref1="CH" ref2="CH”error=“19.9E2”>19.9E3</me:experimentalRate></me:PTpair>

<me:PTpair units="Torr" P="8.2" T= "240" precision="qd"><me:bathGas> He </me:bathGas><me:experimentalRate ref1="CH" ref2="CH”error=“1.80E2”>1.80E3</me:experimentalRate></me:PTpair>
<me:PTpair units="Torr" P="8.2" T= "254" precision="qd"><me:bathGas> He </me:bathGas><me:experimentalRate ref1="CH" ref2="CH”error=“1.75E2”>1.75E3</me:experimentalRate></me:PTpair>
<me:PTpair units="Torr" P="8.2" T= "273" precision="qd"><me:bathGas> He </me:bathGas><me:experimentalRate ref1="CH" ref2="CH”error=“1.82E2”>1.82E3</me:experimentalRate></me:PTpair>

<!--Leeds 2021 -->
<me:PTpair units="Torr" P="34" T= "673" precision="d"> <me:bathGas>He</me:bathGas> <me:experimentalRate ref1="CH" ref2="CH" error=" 1.842E3 "> 1.84E4 </me:experimentalRate> </me:PTpair>
<me:PTpair units="Torr" P="71" T= "673" precision="d"> <me:bathGas>He</me:bathGas> <me:experimentalRate ref1="CH" ref2="CH" error=" 1.74E3 "> 1.74E4 </me:experimentalRate> </me:PTpair>
<me:PTpair units="Torr" P="14.5" T= "673" precision="d"> <me:bathGas>He</me:bathGas> <me:experimentalRate ref1="CH" ref2="CH" error=" 1.85E3 "> 1.85E4 </me:experimentalRate> </me:PTpair>
<me:PTpair units="Torr" P="6.6" T= "673" precision="d"> <me:bathGas>He</me:bathGas> <me:experimentalRate ref1="CH" ref2="CH" error=" 2.23E3 "> 2.23E4 </me:experimentalRate> </me:PTpair>

<me:PTpair units="Torr" P="5.2" T= "673" precision="d"> <me:bathGas>Ar</me:bathGas> <me:experimentalRate ref1="CH" ref2="CH" error=" 2.07E3 "> 2.07E4 </me:experimentalRate> </me:PTpair>
<me:PTpair units="Torr" P="141.5" T= "673" precision="d"> <me:bathGas>Ar</me:bathGas> <me:experimentalRate ref1="CH" ref2="CH" error=" 2.04E3 "> 2.04E4 </me:experimentalRate> </me:PTpair>
<me:PTpair units="Torr" P="19.6" T= "673" precision="d"> <me:bathGas>Ar</me:bathGas> <me:experimentalRate ref1="CH" ref2="CH" error=" 2.14E3 "> 2.14E4 </me:experimentalRate> </me:PTpair>

<me:PTpair units="Torr" P="174" T= "748" precision="d"> <me:bathGas>Ar</me:bathGas> <me:experimentalRate ref1="CH" ref2="CH" error=" 2.62E3 "> 2.62E4 </me:experimentalRate> </me:PTpair>
<me:PTpair units="Torr" P="70" T= "748" precision="d"> <me:bathGas>Ar</me:bathGas> <me:experimentalRate ref1="CH" ref2="CH" error=" 2.37E3 "> 2.37E4 </me:experimentalRate> </me:PTpair>
<me:PTpair units="Torr" P="12.0" T= "748" precision="d"> <me:bathGas>Ar</me:bathGas> <me:experimentalRate ref1="CH" ref2="CH" error=" 2.44E3 "> 2.44E4 </me:experimentalRate> </me:PTpair>

<me:PTpair units="Torr" P="11.5" T= "573" precision="d"> <me:bathGas>Ar</me:bathGas> <me:experimentalRate ref1="CH" ref2="CH" error=" 1.37E3 "> 1.37E4 </me:experimentalRate> </me:PTpair>
<me:PTpair units="Torr" P="27" T= "573" precision="d"> <me:bathGas>Ar</me:bathGas> <me:experimentalRate ref1="CH" ref2="CH" error=" 1.34E3 "> 1.34E4 </me:experimentalRate> </me:PTpair>
<me:PTpair units="Torr" P="100.1" T= "573" precision="d"> <me:bathGas>Ar</me:bathGas> <me:experimentalRate ref1="CH" ref2="CH" error=" 1.44E3 "> 1.44E4 </me:experimentalRate> </me:PTpair>
<me:PTpair units="Torr" P="274.3" T= "573" precision="d"> <me:bathGas>Ar</me:bathGas> <me:experimentalRate ref1="CH" ref2="CH" error=" 1.83E3 "> 1.83E4 </me:experimentalRate> </me:PTpair>

<me:PTpair units="Torr" P="11.5" T= "472" precision="dd"> <me:bathGas>Ar</me:bathGas> <me:experimentalRate ref1="CH" ref2="CH" error=" 0.832E3 "> 0.832E4 </me:experimentalRate> </me:PTpair>
<me:PTpair units="Torr" P="27" T= "472" precision="dd"> <me:bathGas>Ar</me:bathGas> <me:experimentalRate ref1="CH" ref2="CH" error=" 0.955E3 "> 0.955E4 </me:experimentalRate> </me:PTpair>
<me:PTpair units="Torr" P="100.1" T= "472" precision="dd"> <me:bathGas>Ar</me:bathGas> <me:experimentalRate ref1="CH" ref2="CH" error=" 1.08E3 ">1.08E4 </me:experimentalRate> </me:PTpair>
<me:PTpair units="Torr" P="274.3" T= "475" precision="dd"> <me:bathGas>Ar</me:bathGas> <me:experimentalRate ref1="CH" ref2="CH" error=" 1.63E3 ">1.63E4 </me:experimentalRate> </me:PTpair>

<me:PTpair units="Torr" P="10.8" T= "372" precision="dd"> <me:bathGas>Ar</me:bathGas> <me:experimentalRate ref1="CH" ref2="CH" error=" 0.444E3 "> 0.444E4 </me:experimentalRate> </me:PTpair>
<me:PTpair units="Torr" P="35" T= "372" precision="dd"> <me:bathGas>Ar</me:bathGas> <me:experimentalRate ref1="CH" ref2="CH" error=" 0.637E3 "> 0.637E4 </me:experimentalRate> </me:PTpair>
<me:PTpair units="Torr" P="106" T= "372" precision="dd"> <me:bathGas>Ar</me:bathGas> <me:experimentalRate ref1="CH" ref2="CH" error=" 1.08E3 "> 1.08E4 </me:experimentalRate> </me:PTpair>
<me:PTpair units="Torr" P="274.3" T= "372" precision="dd"> <me:bathGas>Ar</me:bathGas> <me:experimentalRate ref1="CH" ref2="CH" error=" 1.86E3 ">1.86E4 </me:experimentalRate> </me:PTpair>

<me:PTpair units="Torr" P="9.0" T= "298" precision="qd"> <me:bathGas>Ar</me:bathGas> <me:experimentalRate ref1="CH" ref2="CH" error=" 0.30E3 ">0.30E4 </me:experimentalRate> </me:PTpair>
<me:PTpair units="Torr" P="30.1" T= "298" precision="qd"> <me:bathGas>Ar</me:bathGas> <me:experimentalRate ref1="CH" ref2="CH" error=" 0.611E3 ">0.611E4 </me:experimentalRate> </me:PTpair>
<me:PTpair units="Torr" P="110.7" T= "298" precision="qd"> <me:bathGas>Ar</me:bathGas> <me:experimentalRate ref1="CH" ref2="CH" error=" 1.50E3 ">1.50E4 </me:experimentalRate> </me:PTpair>
<me:PTpair units="Torr" P="304" T= "298" precision="qd"> <me:bathGas>Ar</me:bathGas> <me:experimentalRate ref1="CH" ref2="CH" error=" 2.95E3 "> 2.95E4 </me:experimentalRate> </me:PTpair>

<me:PTpair units="Torr" P="35" T= "673" precision="d"> <me:bathGas>Ar</me:bathGas> <me:experimentalRate ref1="CH" ref2="CH" error=" 1.83E3 "> 1.83E4 </me:experimentalRate> </me:PTpair>
<me:PTpair units="Torr" P="15" T= "673" precision="d"> <me:bathGas>Ar</me:bathGas> <me:experimentalRate ref1="CH" ref2="CH" error=" 1.76E3 "> 1.76E4 </me:experimentalRate> </me:PTpair>
<me:PTpair units="Torr" P="7.0" T= "673" precision="d"> <me:bathGas>Ar</me:bathGas> <me:experimentalRate ref1="CH" ref2="CH" error=" 1.94E3 "> 1.94E4 </me:experimentalRate> </me:PTpair>
<me:PTpair units="Torr" P="70" T= "673" precision="d"> <me:bathGas>Ar</me:bathGas> <me:experimentalRate ref1="CH" ref2="CH" error=" 1.76E3 "> 1.76E4 </me:experimentalRate> </me:PTpair>

<!--Brownsword 10% error-->

<me:PTpair units="Torr" P="10" T= "744" precision="d"> <me:bathGas>Ar</me:bathGas> <me:experimentalRate ref1="CH" ref2="CH" error=" 2.80E3 "> 2.80E4 </me:experimentalRate> </me:PTpair>
<me:PTpair units="Torr" P="30" T= "744" precision="d"> <me:bathGas>Ar</me:bathGas> <me:experimentalRate ref1="CH" ref2="CH" error=" 2.57E3 "> 2.57E4 </me:experimentalRate> </me:PTpair>
<me:PTpair units="Torr" P="100" T= "744" precision="d"> <me:bathGas>Ar</me:bathGas> <me:experimentalRate ref1="CH" ref2="CH" error=" 2.17E3 "> 2.17E4 </me:experimentalRate> </me:PTpair>
<me:PTpair units="Torr" P="400" T= "744" precision="d"> <me:bathGas>Ar</me:bathGas> <me:experimentalRate ref1="CH" ref2="CH" error=" 2.92E3 "> 2.92E4 </me:experimentalRate> </me:PTpair>

<me:PTpair units="Torr" P="10" T= "584" precision="d"> <me:bathGas>Ar</me:bathGas> <me:experimentalRate ref1="CH" ref2="CH" error=" 1.59E3 "> 1.59E4 </me:experimentalRate> </me:PTpair>
<me:PTpair units="Torr" P="30" T= "584" precision="d"> <me:bathGas>Ar</me:bathGas> <me:experimentalRate ref1="CH" ref2="CH" error=" 1.39E3 "> 1.39E4 </me:experimentalRate> </me:PTpair>

```
<me:PTpair units="Torr" P="100" T= "584" precision="d"> <me:bathGas>Ar</me:bathGas> <me:experimentalRate ref1="CH" ref2="CH" error=" 1.74E3 ">     1.74E4 </me:experimentalRate> </me:PTpair>
<me:PTpair units="Torr" P="400" T= "584" precision="d"> <me:bathGas>Ar</me:bathGas> <me:experimentalRate ref1="CH" ref2="CH" error=" 2.14E3 ">     2.14E4 </me:experimentalRate> </me:PTpair>


<me:PTpair units="Torr" P="10" T= "484" precision="dd"> <me:bathGas>Ar</me:bathGas> <me:experimentalRate ref1="CH" ref2="CH" error=" 0.942E3 ">   0.942E4 </me:experimentalRate> </me:PTpair>
<me:PTpair units="Torr" P="30" T= "484" precision="dd"> <me:bathGas>Ar</me:bathGas> <me:experimentalRate ref1="CH" ref2="CH" error=" 0.968E3 ">   0.968E4 </me:experimentalRate> </me:PTpair>
<me:PTpair units="Torr" P="100" T= "484" precision="dd"> <me:bathGas>Ar</me:bathGas> <me:experimentalRate ref1="CH" ref2="CH" error=" 1.33E3 ">   1.33E4 </me:experimentalRate> </me:PTpair>
<me:PTpair units="Torr" P="400" T= "444" precision="dd"> <me:bathGas>Ar</me:bathGas> <me:experimentalRate ref1="CH" ref2="CH" error=" 2.06E3 ">   2.06E4 </me:experimentalRate> </me:PTpair>

<me:PTpair units="Torr" P="10" T= "364" precision="dd"> <me:bathGas>Ar</me:bathGas> <me:experimentalRate ref1="CH" ref2="CH" error=" 0.429E3 ">     0.429E4 </me:experimentalRate> </me:PTpair>
<me:PTpair units="Torr" P="30" T= "364" precision="dd"> <me:bathGas>Ar</me:bathGas> <me:experimentalRate ref1="CH" ref2="CH" error=" 0.599E3 ">   0.599E4 </me:experimentalRate> </me:PTpair>
<me:PTpair units="Torr" P="100" T= "364" precision="dd"> <me:bathGas>Ar</me:bathGas> <me:experimentalRate ref1="CH" ref2="CH" error=" 1.20E3 ">   1.20E4 </me:experimentalRate> </me:PTpair>
<me:PTpair units="Torr" P="400" T= "364" precision="dd"> <me:bathGas>Ar</me:bathGas> <me:experimentalRate ref1="CH" ref2="CH" error=" 2.61E3 ">   2.61E4 </me:experimentalRate> </me:PTpair>

<me:PTpair units="Torr" P="10" T= "294" precision="qd"> <me:bathGas>Ar</me:bathGas> <me:experimentalRate ref1="CH" ref2="CH" error=" 0.319E3 ">     0.319E4 </me:experimentalRate> </me:PTpair>
<me:PTpair units="Torr" P="30" T= "294" precision="qd"> <me:bathGas>Ar</me:bathGas> <me:experimentalRate ref1="CH" ref2="CH" error=" 0.564E3 ">   0.564E4 </me:experimentalRate> </me:PTpair>
<me:PTpair units="Torr" P="100" T= "294" precision="qd"> <me:bathGas>Ar</me:bathGas> <me:experimentalRate ref1="CH" ref2="CH" error=" 1.35E3 ">   1.35E4 </me:experimentalRate> </me:PTpair>
<me:PTpair units="Torr" P="400" T= "294" precision="qd"> <me:bathGas>Ar</me:bathGas> <me:experimentalRate ref1="CH" ref2="CH" error=" 3.29E3 ">   3.29E4 </me:experimentalRate> </me:PTpair>


<!-- ch3 decompostion via shocj tube -->

<me:PTpair units=“atm”P=“4.116 “  T= “2706” precision="d"><me:bathGas>Ar</me:bathGas><me:experimentalRate ref1=“CH3” ref2=“CH” error=“2214.30718”>14762.04786</me:experimentalRate></me:PTpair>
<me:PTpair units=“atm”P=“1.087 “  T= “2709” precision="d"><me:bathGas>Ar</me:bathGas><me:experimentalRate ref1=“CH3” ref2=“CH”    error=“658.2380484”>4388.253656</me:experimentalRate></me:PTpair
<me:PTpair units=“atm”P=“3.829 “  T= “2717” precision="d"><me:bathGas>Ar</me:bathGas><me:experimentalRate ref1=“CH3” ref2=“CH”    error=“2066.878713”>13779.19142</me:experimentalRate></me:PTpair>
<me:PTpair units=“atm”P=“1.095 “  T= “2738” precision="d"><me:bathGas>Ar</me:bathGas><me:experimentalRate ref1=“CH3” ref2=“CH”    error=“777.7127203”>5184.751469</me:experimentalRate></me:PTpair>
<me:PTpair units=“atm”P=“2.391 “  T= “2763” precision="d"><me:bathGas>Ar</me:bathGas><me:experimentalRate ref1=“CH3” ref2=“CH”error=“1842.638999”>12284.25999</me:experimentalRate></me:PTpai
<me:PTpair units=“atm”P=“1.871 “  T= “2770” precision="d"><me:bathGas>Ar</me:bathGas><me:experimentalRate ref1=“CH3”ref2="CH"error=“1570.338534”>10468.92356</me:experimentalRate></me:PTpair
<me:PTpair units=“atm”P=“4.208 “  T= “2783” precision="d"><me:bathGas>Ar</me:bathGas><me:experimentalRate ref1=“CH3”ref2="CH"error=“3416.735467”>22778.23644</me:experimentalRate></me:PTpair
<me:PTpair units=“atm”P=“3.636 “  T= “2789” precision="d"><me:bathGas>Ar</me:bathGas><me:experimentalRate ref1=“CH3”ref2="CH"error=“3030.92109”>20206.1406</me:experimentalRate></me:PTpair>
<me:PTpair units=“atm”P=“1.883 “  T= “2789” precision="d"><me:bathGas>Ar</me:bathGas><me:experimentalRate ref1=“CH3”ref2="CH"error=“1657.66108”>11051.07387</me:experimentalRate></me:PTpair>
<me:PTpair units=“atm”P=“2.742 “  T= “2802” precision="d"><me:bathGas>Ar</me:bathGas><me:experimentalRate ref1=“CH3” ref2=“CH”error=“2721.603037”>18144.02024</me:experimentalRate></me:PTpair>

<me:PTpair units=“atm”P=“1.042 “  T= “2837” precision="d"><me:bathGas>Ar</me:bathGas><me:experimentalRate ref1=“CH3” ref2=“CH”error=“1416.518439”>9443.456262</me:experimentalRate></me:PTpair>
<me:PTpair units=“atm”P=“2.637 “  T= “2845” precision="d"><me:bathGas>Ar</me:bathGas><me:experimentalRate ref1=“CH3” ref2=“CH”error=“3393.464076”>22623.09384</me:experimentalRate></me:PTpair>
<me:PTpair units=“atm”P=“1.835 “  T= “2848” precision="d"><me:bathGas>Ar</me:bathGas><me:experimentalRate ref1=“CH3” ref2=“CH”error=“2057.922”>13719.48</me:experimentalRate></me:PTpair>
<me:PTpair units=“atm”P=“0.976 “  T= “2858” precision="d"><me:bathGas>Ar</me:bathGas><me:experimentalRate ref1=“CH3” ref2=“CH”error=“1472.867218”>9819.114785</me:experimentalRate></me:PTpair>
<me:PTpair units=“atm”P=“1.907 “  T= “2861” precision="d"><me:bathGas>Ar</me:bathGas><me:experimentalRate ref1=“CH3” ref2=“CH”error=“2288.260237”>15255.06824</me:experimentalRate></me:PTpair>
<me:PTpair units=“atm”P=“3.58 “  T= “2925” precision="d"><me:bathGas>Ar</me:bathGas><me:experimentalRate ref1=“CH3” ref2=“CH”error=“5810.641603”>38737.61068</me:experimentalRate></me:PTpair>
<me:PTpair units=“atm”P=“0.974 “  T= “2944” precision="d"><me:bathGas>Ar</me:bathGas><me:experimentalRate ref1=“CH3” ref2=“CH”error=“1818.683657”>12124.55771</me:experimentalRate></me:PTpair>
<me:PTpair units=“atm”P=“1.838 “  T= “2949” precision="d"><me:bathGas>Ar</me:bathGas><me:experimentalRate ref1=“CH3” ref2=“CH”error=“3900.126683”>26000.84455</me:experimentalRate></me:PTpair>
<me:PTpair units=“atm”P=“3.923 “  T= “2982” precision="d"><me:bathGas>Ar</me:bathGas><me:experimentalRate ref1=“CH3” ref2=“CH”error=“8260.836059”>55072.2404</me:experimentalRate></me:PTpair>
<me:PTpair units=“atm”P=“1.005 “  T= “2984” precision="d"><me:bathGas>Ar</me:bathGas><me:experimentalRate ref1=“CH3” ref2=“CH”error=“2729.55322”>18197.02147</me:experimentalRate></me:PTpair>
<me:PTpair units=“atm”P=“1.094 “  T= “3011” precision="d"><me:bathGas>Ar</me:bathGas><me:experimentalRate ref1=“CH3” ref2=“CH”error=“2767.00587”>18446.7058</me:experimentalRate></me:PTpair>
<me:PTpair units=“atm”P=“0.945 “  T= “3161” precision="d"><me:bathGas>Ar</me:bathGas><me:experimentalRate ref1=“CH3” ref2=“CH”error=“4579.435235”>30529.56823</me:experimentalRate></me:PTpair>
<me:PTpair units=“atm”P=“1.027 “  T= “3198” precision="d"><me:bathGas>Ar</me:bathGas><me:experimentalRate ref1=“CH3” ref2=“CH”error=“5337.881288”>35585.87525</me:experimentalRate></me:PTpair>
<me:PTpair units=“atm”P=“1.024 “  T= “3230” precision="d"><me:bathGas>Ar</me:bathGas><me:experimentalRate ref1=“CH3” ref2=“CH”error=“5682.858919”>37885.72613</me:experimentalRate></me:PTpair>
<me:PTpair units=“atm”P=“1.013 “  T= “3273” precision="d"><me:bathGas>Ar</me:bathGas><me:experimentalRate ref1=“CH3” ref2=“CH”error=“6993.784899”>46625.23266</me:experimentalRate></me:PTpair>
<me:PTpair units=“atm”P=“1.079 “  T= “3348” precision="d"><me:bathGas>Ar</me:bathGas><me:experimentalRate ref1=“CH3” ref2=“CH”error=“8648.055033”>57653.70022</me:experimentalRate></me:PTpair>
<me:PTpair units=“atm”P=“1.039 “  T= “3393” precision="d"><me:bathGas>Ar</me:bathGas><me:experimentalRate ref1=“CH3” ref2=“CH”error=“10346.12147”>68974.14313</me:experimentalRate></me:PTpair>
<me:PTpair units=“atm”P=“1.04 “  T= “3472” precision="d"><me:bathGas>Ar</me:bathGas><me:experimentalRate ref1=“CH3” ref2=“CH”error=“12691.23032”>84608.20215</me:experimentalRate></me:PTpair>
<me:PTpair units=“atm”P=“0.964 “  T= “3527” precision="d"><me:bathGas>Ar</me:bathGas><me:experimentalRate ref1=“CH3” ref2=“CH”error=“14044.88576”>93632.57172</me:experimentalRate></me:PTpair>
<me:PTpair units=“atm”P=“1.005 “  T= “3527” precision="d"><me:bathGas>Ar</me:bathGas><me:experimentalRate ref1=“CH3” ref2=“CH”error=“14642.23048”>97614.8699</me:experimentalRate></me:PTpair>


<!-- <me:PTpair units=“atm”P=“1.425 “ T=“2780” precision="d"><me:bathGas>ArUSA</me:bathGas><me:experimentalRate ref1=“CH3”ref2="CH2"error=“1.63E+03">5420.22308</me:experimentalRate></me:PTpair> -->
<!-- <me:PTpair units=“atm”P=“1.529 “ T=“2665” precision="d"><me:bathGas>ArUSA</me:bathGas><me:experimentalRate ref1=“CH3”ref2="CH2"error=“8.99E+02”>2995.985959</me:experimentalRate></me:PTpair> -->
<!-- <me:PTpair units=“atm”P=“1.6 “ T=“2562” precision="d"><me:bathGas>ArUSA</me:bathGas><me:experimentalRate ref1=“CH3”ref2="CH2"error=“6.00E+02”>1999.205158</me:experimentalRate></me:PTpair> -->
<!-- <me:PTpair units=“atm”P=“1.754 “ T=“2253” precision="d"><me:bathGas>ArUSA</me:bathGas><me:experimentalRate ref1=“CH3”ref2="CH2"error=“5.57E+01”>185.506244</me:experimentalRate></me:PTpair> -->
<!-- <me:PTpair units=“atm”P=“1.488 “ T=“2747” precision="d"><me:bathGas>ArUSA</me:bathGas><me:experimentalRate ref1=“CH3”ref2="CH2"error=“1.35E+03”>4511.659849</me:experimentalRate></me:PTpair> -->
<!-- <me:PTpair units=“atm”P=“1.198 “ T=“2843” precision="d"><me:bathGas>ArUSA</me:bathGas><me:experimentalRate ref1=“CH3”ref2="CH2"error=“1.34E+03”>4455.813973</me:experimentalRate></me:PTpair> -->
<!-- <me:PTpair units=“atm”P=“1.242 “ T=“2698” precision="d"><me:bathGas>ArUSA</me:bathGas><me:experimentalRate ref1=“CH3”ref2="CH2"error=“6.19E+02”>2063.785403</me:experimentalRate></me:PTpair> -->
<!-- <me:PTpair units=“atm”P=“1.288 “ T=“2693” precision="d"><me:bathGas>ArUSA</me:bathGas><me:experimentalRate ref1=“CH3”ref2="CH2"error=“6.22E+02”>2074.916236</me:experimentalRate></me:PTpair> -->
<!-- <me:PTpair units=“atm”P=“1.285 “ T=“2550” precision="d"><me:bathGas>ArUSA</me:bathGas><me:experimentalRate ref1=“CH3”ref2="CH2"error=“4.46E+02”>1485.427957</me:experimentalRate></me:PTpair> -->
<!-- <me:PTpair units=“atm”P=“1.289 “ T=“2417” precision="d"><me:bathGas>ArUSA</me:bathGas><me:experimentalRate ref1=“CH3”ref2="CH2"error=“1.38E+02”>459.6396289</me:experimentalRate></me:PTpair> -->
<!-- <me:PTpair units=“atm”P=“1.37 “ T=“2375” precision="d"><me:bathGas>ArUSA</me:bathGas><me:experimentalRate ref1=“CH3”ref2="CH2"error=“1.17E+02”>389.374138</me:experimentalRate></me:PTpair> -->
<!-- <me:PTpair units=“atm”P=“1.161 “ T=“2941” precision="d"><me:bathGas>ArUSA</me:bathGas><me:experimentalRate ref1=“CH3”ref2="CH2"error=“2.15E+03”>7176.37548</me:experimentalRate></me:PTpair> -->
<!-- <me:PTpair units=“atm”P=“1.409 “ T=“2276” precision="d"><me:bathGas>ArUSA</me:bathGas><me:experimentalRate ref1=“CH3”ref2="CH2"error=“5.43E+01”>181.1399892</me:experimentalRate></me:PTpair> -->
<!-- <me:PTpair units=“atm”P=“3.698 “ T=“2765” precision="d"><me:bathGas>ArUSA</me:bathGas><me:experimentalRate ref1=“CH3”ref2="CH2"error=“2.71E+03”>9027.799818</me:experimentalRate></me:PTpair> -->
<!-- <me:PTpair units=“atm”P=“3.626 “ T=“2882” precision="d"><me:bathGas>ArUSA</me:bathGas><me:experimentalRate ref1=“CH3”ref2="CH2"error=“4.35E+03”>14488.5587</me:experimentalRate></me:PTpair> -->
<!-- <me:PTpair units=“atm”P=“3.898 “ T=“2587” precision="d"><me:bathGas>ArUSA</me:bathGas><me:experimentalRate ref1=“CH3”ref2="CH2"error=“1.10E+03”>3677.64279</me:experimentalRate></me:PTpair> -->
<!-- <me:PTpair units=“atm”P=“2.988 “ T=“2953” precision="d"><me:bathGas>ArUSA</me:bathGas><me:experimentalRate ref1=“CH3”ref2="CH2"error=“7.30E+03”>24330.41221</me:experimentalRate></me:PTpair> -->
<!-- <me:PTpair units=“atm”P=“2.991 “ T=“2635” precision="d"><me:bathGas>ArUSA</me:bathGas><me:experimentalRate ref1=“CH3”ref2="CH2"error=“1.40E+03”>4669.58587</me:experimentalRate></me:PTpair> -->
<!-- <me:PTpair units=“atm”P=“1.191 “ T=“2707” precision="d"><me:bathGas>ArUSA</me:bathGas><me:experimentalRate ref1=“CH3”ref2="CH2"error=“7.83E+02”>2609.766454</me:experimentalRate></me:PTpair> -->
<!-- <me:PTpair units=“atm”P=“1.091 “ T=“2975” precision="d"><me:bathGas>ArUSA</me:bathGas><me:experimentalRate ref1=“CH3”ref2="CH2"error=“2.98E+03”>9933.530274</me:experimentalRate></me:PTpair> -->
<!-- <me:PTpair units=“atm”P=“1.119 “ T=“2871” precision="d"><me:bathGas>ArUSA</me:bathGas><me:experimentalRate ref1=“CH3”ref2="CH2"error=“1.45E+03”>4827.110509</me:experimentalRate></me:PTpair> -->
<!-- <me:PTpair units=“atm”P=“1.17 “ T=“2790” precision="d"><me:bathGas>ArUSA</me:bathGas><me:experimentalRate ref1=“CH3”ref2="CH2"error=“1.11E+03”>3705.405366</me:experimentalRate></me:PTpair> -->



  </me:PTs>
</me:conditions>

<me:modelParameters>
  <!--Specify grain size directly...-->
  <me:grainSize units="cm-1">35</me:grainSize>
  <!--...or by the total number of grains
     <me:numberOfGrains> 500 </me:numberOfGrains>-->
  <!-- Specify increased energy range -->
     <!-- <me:maxTemperature>6000</me:maxTemperature> -->
  <me:automaticallySetMaxEne>1.0e-15</me:automaticallySetMaxEne>
   <!-- <me:energyAboveTheTopHill>25.0</me:energyAboveTheTopHill>  -->
```

```
  </me:modelParameters>

  <me:control>
    <me:calcMethod xsi:type="me:marquardt">
      <me:MarquardtIterations>10</me:MarquardtIterations>
      <me:MarquardtTolerance>0.000001</me:MarquardtTolerance>
      <me:MarquardtDerivDelta>.0025</me:MarquardtDerivDelta>
    </me:calcMethod>
    <me:printSpeciesProfile />
    <me:testRateConstants />
    <!--<me:printGrainDOS />
    <me:printGrainkfE />
    <me:printGrainkbE />-->

    <!-- <me:ForceMacroDetailedBalance/> -->
    <!-- <me:calcMethod xsi:type="me:marquardt"> -->
    <!-- <me:MarquardtIterations>15</me:MarquardtIterations> -->
    <!-- <me:MarquardtTolerance>1e-8</me:MarquardtTolerance> -->
    <!-- <me:MarquardtDerivDelta>0.025</me:MarquardtDerivDelta> -->
    <!-- </me:calcMethod> -->

    <!-- <me:testDOS/> -->
    <me:testMicroRates />
    <me:testRateConstant />
    <!-- <me:printGrainDOS /> -->
    <!-- <me:printCellDOS /> -->
    <!-- <me:printReactionOperatorColumnSums /> -->
    <!-- <me:printTunnellingCoefficients /> -->
    <me:printGrainkfE />
    <!-- <me:printGrainBoltzmann /> -->
    <me:printGrainkbE />
    <me:eigenvalues>10</me:eigenvalues>

  </me:control>

</me:mesmer>
```


1. Brownsword, R. A.; Canosa, A.; Rowe, B. R.; Sims, I. R.; Smith, I. W. M.; Stewart, D. W. A.; Symonds, A. C.; Travers, D., Kinetics over a wide range of temperature (13-744 K): Rate constants for the reactions of CH(v=0) with H_2 and D_2 and for the removal of CH(v=1) by H_2 and D_2. *J. Chem. Phys.* **1997,** *106* (18), 7662-7677.

Paul Seakins: p.w.seakins@leeds.ac.uk
Mark Blitz: m.blitz@leeds.ac.uk
